# Assessment of COVID-19 preparedness response plan on higher education students simulation of WHO intra-action review in Egypt

**DOI:** 10.1038/s41598-023-27713-1

**Published:** 2023-01-13

**Authors:** Hager Moustafa Saeed, Azza SAAD ELGHAREEB, Mostafa Abdel Aziz El-Hodhod, Gamal Samy

**Affiliations:** 1 October 6 University, 6th of October City, Giza, Egypt; 2 Faculty of Medicine, October 6 University, 6th of October City, Giza, Egypt; 3 Faculty of Medicine, October 6 University, 6th of October City, Giza, Egypt

**Keywords:** Health care, Health occupations, Risk factors

## Abstract

Because of the COVID-19 outbreak, Mass gathering restrictions were imposed. The lockdown of the Higher Education Institutions was obligatory to save lives. In February 2021 in Egypt, HEIs were allowed to ease the lockdown restrictions on a case-by-case basis gradually. In this paper, we propose a risk evaluation of planned regular mass gathering events during the pandemic, such as students gathering on-campus during indoor exams, by implementing WHO COVID-19 Strategic Preparedness and Response Plan through Intra-Action Review guidance. This one-group posttest-only design study was done on October 6 University campus during indoor students' exams in Giza, Egypt. We conducted IAR to implement the WHO's COVID- 19 SPRP; Country-level coordination; risk communication; surveillance, rapid response teams; points of entry; infection prevention control; laboratories; supply chain; case management; essential health services, and other possible topics. Between February-21, 2021; April-10, 2021, 25,927 students attended the on-campus living exams. Our result suggests that the high level of Readiness–Capacity during mass gatherings will reduce COVID-19 transmission. The most compelling evidence is the significance of synchronization between the ten pillars in preventing COVID-19 transmission. These findings may be used to influence decision-making for continual improvement of the operational planning guidelines during the outbreaks.

## Introduction

COVID-19 mortality rates in Africa were expected to be greater than elsewhere, even among youngsters. The first African country to report a case of COVID-19 was Egypt in February 2020^1^. And so, the lockdown of the Higher Education Institutions (HEIs) was obligatory to save lives^2^. Mass gathering restrictions were imposed, including live indoors on-campus classes. The decision was made to hold the study on campus^2^. In February 2021 in Egypt, Higher Education Institutions were allowed to gradually ease the lockdown restrictions on a case-by-case basis.

Several observations imply that an event's capacity for spreading infectious diseases is determined by the sort of event; the risk of SARS-CoV-2 transmission associated with specific events or conferences is less than others^3,4^.

In the consequences of the COVID-19 outbreak, the WHO outlined the transmission routes and scenario of COVID-19 and released the COVID-19 Strategic Preparedness and Response Plan guideline structured in the critical ten public health pillars^5^.

And so, WHO published the Operational Planning Guideline in accordance with the SPRP 2021 to provide institutions with high-level actions on each of the ten pillars which can be implemented at the sub-national level^6^. The implementation of the guideline was evaluated by (the operational planning guideline monitor and evaluation framework 2021–2022), which included Key Performance Indicators (KPIs) for each pillar checklist with the recommended actions under Strategic Preparedness and Response Plan (2020: 2021)^7^. All the operational planning policies and procures were reviewed and revised to accommodate inclusive learning^8^, students with disabilities^9^, gender equality, equity, and human rights, and in compliance with twelve goals of the United Nations Sustainable Development Goals^10^.

The International Health Regulations (2005) monitoring and evaluation framework encouraged the decision-makers to conduct After-Action Review (AAR) after the official declaration by the end of public health events^11^. In the context of COVID-19, WHO updated a guide for conducting Intra-Action Reviews (IARs) to improve the crucial sustainable response during the pandemic^12^. The main objective of IAR is to enable proactive response and continual improvement in real-time. In contrast to AAR which is conducted post-emergency^13^.

The Intra-Action Review guidance is lined up with the WHO COVID-19 Strategic Preparedness and Response Plan and in accordance with the transmission scenario^14^. This guidance reviews the ten public health pillars responses, and preparedness in the phase of the Pre-IAR, During the IAR, and post the IAR^12^.

In this paper, we propose a risk evaluation of planned regular mass gathering events during the pandemic, such as students gathering on-campus during indoor exams in Higher Education Institutions, by implementing the WHO Mass Gatherings COVID-19 risk assessment tool and the operational planning guideline of COVID-19 SPRP through WHO IAR guidance template.

## Methods

### Study design

The study is a *one-group posttest-only design* of an exposure model for preventing SARS-CoV-2 transmission among students during the on-campus exams^15^.

*The study was done on* October 6 University *campus during indoor students' exams* on February 23, 2021, in Giza, Egypt. Participants were age group 15‑24 years^7^. The study included the students who were verified to be eligible to attend the exams, had no COVID-19 symptoms, nor contact case, and had a negative PCR test within three days before exams.

Research Ethics Committee of October 6 University approved the study protocol from an ethical point of view. Approval number: PR-De-2110003. All participants provided written informed consent. All methods were performed in accordance with October 6 University guidelines and regulations.

### Procedure

We conducted Intra Action Review based on WHO IARs guidance 10 ready-to-use and customizable accompanying tools. A database of over 300 COVID-19-specific trigger questions was answered^12^.

The IAR roadmap process is divided into three phases, (pre, during, and post). The pre-IAR phase^12^, started one week before the exams. We used the WHO Mass Gathering COVID-19 Risk Assessment Tool–Generic Events, the tool identifies the numerical score of the risk factor and assigned the mitigation measure during the event. The final overall score was added to the decision matrix to obtain a risk category (e.g., very low, low, moderate, or very high)^4^.

According to the risk category, A crosscutting review of the WHO's ten pillars was conducted. The ten pillars included in the review were: Country-level coordination; risk communication; surveillance, rapid response teams; points of entry; infection prevention control; laboratories; supply chain; case management; essential health services, and other possible topics.

During the IAR, we used readiness–capacity key performance indicators for constant monitoring and evaluation of the SOPs during the IAR^14^.

Post-IAR included the results and follow-up. The IAR feedback template was gathered from all the participants. We documented the pre, during, and post-actions in the WHO IAR template form^12^.

### Objectives

The objectives of the IAR were to identify best practices, restricting variables, and challenges, strengths and gaps in the university's COVID-19 response on the sub-national level and document all of these using WHO IAR notetaking templates for long-term actions.

This IAR will influence the Higher Education Providers and multi-sector stakeholder in establishing a resilient Higher Education System which accommodate any further outbreaks.

The primary outcome was the number of positive SARS-CoV-2 PCR tests during the exams till one-week post-exam gathering from the illegible students. The secondary outcome was to assess the efficacy of the Strategic Preparedness and Response Plan on preventing COVID-19 transmission during on-campus exams regularly using Readiness–Capacity KPIs. The anticipated cut-off of the KPIs score (is 85–80%)^16^.

### Statistical analysis

Data were statistically described in terms of mean ± standard deviation (± SD), median and range, or frequencies (number of cases) and percentages when appropriate. Correlation between the total KPIs score and the infection rate was done using Pearson moment correlation. All statistical calculations were done using the computer program IBM SPSS (Statistical Package for Social Science; IBM Corp, Armonk, NY, USA) release 22 for Microsoft Windows.

## Results

This study includes the 14 faculties of the university. Between (February 21, 2021, to April 10, 2021), 25.927 Students attended the on-campus living exams. All the students presented negative PCR results for SARS-CoV-2 conducted within 3 days preceding the exams.

Stakeholders included the Higher Education Providers, MOH, Ministry of Higher Education, Incident Management System (IOM), Egypt Emergency Operations Centre (EOC), Disaster Risk and Reduction Management (DRR), University President Office, Point of Entry, Ministry of Interior, Ministry of Defense, Ministry of Transportation, Egyptian Authority for Unified Procurement (UPA)and other relevant stakeholders.

The IAR addressed ten COVID-19 public health response pillars in the university's reopening following a ten-months lockdown due to COVID-19 quarantine. Key strengths of this IAR include proactive communication on the subnational level in the university that enhance multisectoral coordination at all levels with the involvement of civilian-military; development of a COVID-19 response plan in Higher Education sectors, founding of COVID-19 guidelines for resilience Higher Education System; and organization of Integrated Hospital Referral System and a COVID-19 laboratory network.

We highlighted the crucial points that were represented in the O6U IAR report to be key lessons learned.

*Pillar 1, Country-level coordination*, we coordinated the university Incident Management System (IMS) in collaboration with the country's Emergency Operations Centre (EOC) on the sub-national level. We identified stakeholders who would be engaged in analyzing the COVID-19 SPRP, as well as the operational guidelines, and then held a meeting to discuss the multisectoral coordination, risk communication, community engagement (C3), and standard operating procedures (SOPs) with stakeholders. Lack of multi-sectoral communication at all levels resulted in delays in the response and improper coordination in the de-escalation of the emergency.

*Pillar 2; Risk Communication and Community Engagement (RCCE),* The RCCE team used the Platforms for risk communication and rumors management (University website, hotlines, student union groups, campaigns, social media)^17^.

The RCCE team organized regular campaigns on Public Health and Social Measures PHMS. Hygiene protocols and WASH were emphasized in the campaigns^18^. Visual reminders of basic preventive measures, especially physical distancing, face masks, hand hygiene practices, and cough etiquette were used^3^.

The main challenge was to communicate the transmission classification to the university community and to encourage the students to adhere to the PHSMs. The long-term recommendation was the use of the rumor log-in documentation to assure proactive RCCE.

*Pillar 3, Surveillance, case investigation, and contact tracing*. Case and contact definitions were based on WHO guidance on global surveillance for COVID-19 in humans^19^. The surveillance system was established. Even the majority of the population in the university healthy young people, superspreading consequences could vary depending on the demographic which is indicating a low risk of transmission^20^. According to WHO Surveillance and Case Definition Guidance, participants' ages ranged from 15 to 24^21^.

The Rapid Response Team legal framework (RRTs) and the contact tracing process were authorized. The number of new confirmed cases was recorded daily. All the data were documented in the WHO case investigation template^12^. The RRT leader reports the incident event to IAR university officials. We used the Key Performance Indicators for monitoring SOPs regularly which included active case finding and contact tracing, IPCs, occupational health, and hazard, communicating risk, and managing rumors^22^. Conducting contact tracing system and overcoming concealment was one of the challenges in the surveillance system. The recommendation was to facilitate the obstacles of the RRT legal framework.

*Pillar 4, Points of entry, international travel and transport, and mass gatherings*. We declared the emergency contingency plans, as well as hospital referral guidelines. Safe entry and transportation guidelines and Process mapping flow charts were released on the identified platform^23^.

We identified one gate for each faculty. The gates were sorted according to Value stream mapping (VSM) analysis results^24^. Advanced screening (thermal thermometers, infrared scanners) and IPC measures were applied at each gate^25^.

The RRT monitored and supervised the student’s entrance and triage methodologies to the exams committees and assuring post-exams evacuation routes without any obstacles.

All students were instructed to transmit their PCR results. Rapid Antigen Diagnostic Tests (RADTs) were used as a screening tool following assessments^26^.

The difficulties in Managing the students crowding at the entrance and monitoring Social Measures PHMS in the POEs were due to a lack of preparedness and lack of cooperation with other stakeholders.

*Pillar 5, Adherence to IPC measures were implemented in the SOPs*^6^. The IPC operational planning guideline encompassed PHSMs (public health and social measures), including personal protective measures (e.g., avoiding crowded settings, physical distancing, respiratory etiquette, WASH, hand hygiene, and mask-wearing), environmental measures (e.g., disinfection, cleaning, and ventilation), surveillance and response measures (e.g., testing, contact tracing, quarantine, and isolation), physical distancing measures (e.g., regulating the flow and number of student attending exams, maintaining distance, and monitoring the lead time of the students through the value stream mapping)^27^. The best practices were the cleaning and disinfection of all the classrooms and the facilities. Also, disinfection of the buses on a regular basis four times after each cycle. The significant challenge was the reduction in the number of the IPC team. Moreover, the prolonged lead time for the disinfectant and PPE equipment.

*Pillar 6, National laboratory system* We used the Rapid Antigen Diagnostic Tests (RADTs) as a screening tool following assessments^26^. For suspected and confirmed cases, there were SOPs for cooperation and collaboration with national stakeholders and the Ministry of Health^6^.

The IAR committee specified specific labs for the COVID-19 rapid test and implement the IPCs in these laps. The ventilation system in the labs is based on three types of ventilation^28^.

*Pillar 7, Case management and knowledge sharing* According to case management operating procedures, we organized an Integrated Hospital Referral System for students with positive RADTs with the help of National authorities where needed^6^. Students with positive RADTs were referred to Ministry of health to receive the treatment protocol.

*Pillar 8, Operational support and logistics in the management of supply chains and the workforce* We conducted facilities needs and surge capacity analyses, considering that each amphitheater will be only half capacity during exams. The University campus covers 170,000 m2, with landscapes accounting for more than half of it. We evaluated the available space and floor plans, interior elevations considering half capacity in each building section, stacking, and blocking diagrams, amphitheaters, bathrooms, exterior elevations, and student transportation at the university. The ventilation rate was calculated for each building. The university depended on a hybrid ventilation system to achieve the targeted ventilation rate^28^. All the buildings (corridors, amphitheaters, classrooms, staircases) had three types of ventilation (natural, mechanical, and hybrid). The classrooms were provided by High-Efficiency Particulate Air (HEPA)^28^.

We established *Five Event Medical Services (EMSs) based on surge capacity and risk assessment results*. The clinics had a private entrance and exit far away from the main gate building^18^.

Regarding supply chain management, we devised a PPE supply chain process and compiled a list of the most important supplies. We used the WHO COVID-19 Essential Supplies Forecasting Tool^29^.

We examined scaling up operations and logistics in the emergency prospective and retrospective examination of the PPE supplies and suppliers in accordance with the standard after identifying the significant risk in the supply chain (infection, replacement, temperature changes, warehouse facilities).

There was only one supplier for some PPE items which may cause a shortage and affect the logistics of service delivery. Other essential supplies were secured in collaboration with Egyptian Authority for Unified Procurement (UPA).

The recommended action is to reduce the number of zero-stock items and identify more than one supplier per category.

*Pillar 9; Maintaining essential health services during the COVID-19 outbreak*, The university hospital encompasses 320 beds. The population of the hospital catchment area is approximately 20,000 people. To avoid morbidity and mortality, all healthcare services were provided in the hospital as usual considering workforce resiliency. This includes ongoing care for comorbidities particularly since comorbid and older individuals are at a higher risk of contracting COVID-19 and dying from it

The university hospital-maintained Women's center services and Sexual Health Reproductive clinics during the COVID-19 outbreak. According to SPRP, Vaccination services, as well as clinics for communicable and non-communicable diseases, were working 24 h^6^.

*Pillar 10, other possible topics* All policies, plans, and measures considered gender, equity, and disadvantaged subpopulations (people with disabilities, persons with low socioeconomic status, and others experiencing exclusion and discrimination)^10,30^.

**Figure 1:**
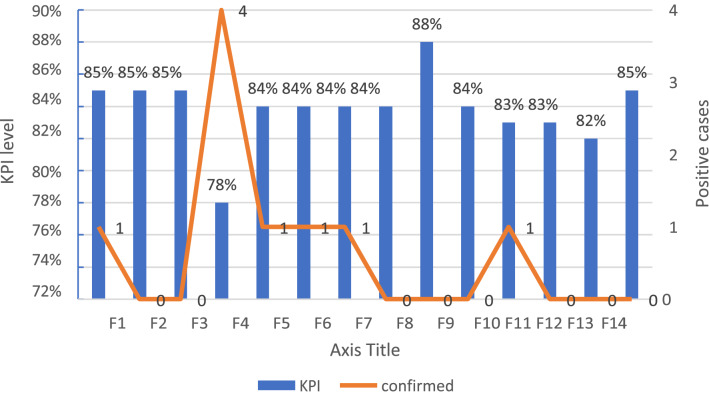
Shows each faculty's Readiness–Capacity KPIS score and the distribution of confirmed cases (N = 9): The blue columns represent the faculty's Readiness–Capacity KPIS score. The distribution of COVID-19 cases among the faculties is represented by the orange line.

23 students were admitted to the *Five Event Medical Services* throughout the tests. Nine of those students had positive PCR. Four of them were from one faculty (Fig. 1)

According to the 14 faculty's Readiness–Capacity KPIS level, 92.8% of them met 80% of the KPIs. This faculty had a 56% infection transmission rate. Only 7.2% of faculties failed to meet the target, with a 44% infection transmission rate.

**Figure 2:**
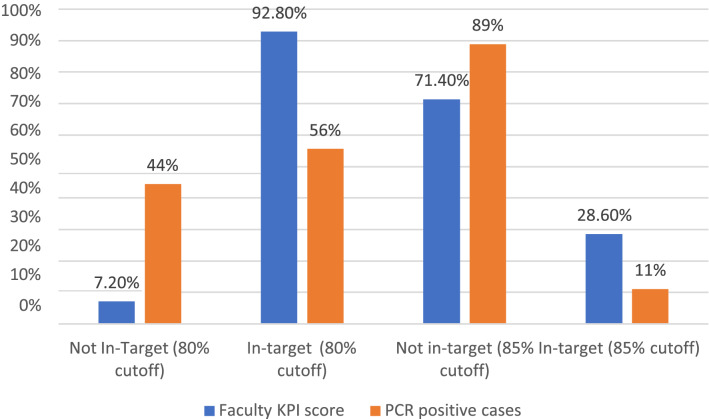
The faculties in general' Readiness–KPIs for capacity and infection transmission rate. The blue columns represent the overall faculties Readiness–Capacity KPIS level, which ranges from 80 to 85%. The infection transmission rate on each level is represented by the orange columns.

Approximately 28.6% of faculties met the 85% Readiness–Capacity KPIs target. In this faculty, the infection transmission rate was 11%. The target was not met by 71.4% of faculties, with an infectious transmission rate of 89% in this faculty (Fig. 2).

The number of positive cases increased substantially during the exams; three cases were identified on March 11th. These cases were from the faculty with the lowest KPI (78%).

Table 1 demonstrates that 8 of the 9 positive confirmed cases were from faculties that were not in the target, while one case was from a target faculty.

**Table 1:** Shows the infection incidence among probable causes based on faculty KPIs (N = 23).

PCR results	In-target (85% cutoff)		Total
	Not in-target	In-target	
Confirmed			
Negative			
Count	11	3	14
% within in-target (85% cutoff)	57.9%	75.0%	60.9%
Positive			
Count	8	1	9
% within in-target (85% cutoff)	42.1%	25.0%	39.1%
Total			
Count	19	4	23
% within in-target (85% cutoff)	100.0%	100.0%	100.0%

## Discussion

To our knowledge, this was the first IAR in HEIs to review the implementation of COVID-19 SPRP. The privileged of conducting IAR is to build a sustainable education system with a resilient ability to provide critical ongoing response and continual operation improvements, as well as challenges from the perspective of participants, stakeholders, students, and Higher Education Providers during the COVID-19 pandemic.

This study revealed a variety of successful practices; however, it also identifies the significant obstacles during indoor students' exams on the university campus to strengthen COVID-19 response in HEIs.

The systemic report of the IAR elaborated that students gathering on-campus during indoor exams in Higher Education Institutions during the COVID-19 outbreaks considering the level of Readiness–Capacity and adhering to COVID-19 SPRP could counteract the transmission of COVID-19.

The IAR road map started one week before the exams till two weeks after the exams from February 21, 2021, to April 10, 2021. The IAR roadmap encompasses pre-IAR, during-IAR, and post-IAR^31^. The Pre-IAR phase was initiated with the WHO Mass Gathering Risk Assessment Tool^4^. The context of the WHO Mass Gatherings COVID-19 risk assessment tool reflects the event's risk factors as well as the producers' ability to mitigate these factors during the COVID-19 outbreak^4^. According to the number of participants, surge capacity, geographical distribution, and the students’ compliance with hygiene protocols, the overall risk of transmission and further spread of COVID-19 was considered very low^4^.

The IAR reviewed the 10 domains of the COVID-19 preparedness and response pillars. WHO identified a target level for each pillar in the COVID-19 SPRP guidance and illustrated that the targets are dynamic and may raise to 100% after a few months^7^. The importance of the overall average target was to evaluate the Readiness–Capacity level of the ten pillars of operational guidelines in a balance. Depending on the nature of the event and surge capacity we classify the overall average target of the pillars as 80% and 85%^7^.

On the 80% of Readiness–Capacity KPIs level, the infection transmission rate was 56% in the faculties that achieved the KPI target. While on the 85% of Readiness–Capacity KPIS level, the infection transmission rate was 11% in the faculties that achieved the KPI target. This serves as a major proof of the correlation between Readiness–Capacity KPIs level and the transmission of infection rate. We found that most confirmed cases were from faculties that did not meet the minimum Readiness–Capacity KPIS level (80%). This indicates the impact of the Readiness–Capacity level on the risk of transmission of COVID-19.

*Pillar 1, Country-level coordination*, and multi-sectoral risk communication are essential to outline the internal and external concerns to be addressed during the on-campus exams. All the stakeholders were involved in the risk assessment process and the SPRP. The IAR emphasized the multisectoral aspects of responding to COVID-19 and revealed the challenges faced by the university. This collaboration enhanced the well-established 3C and reduce the gaps across all the involved parties^3^. To avoid any delays in response, proactive coordination between the IMS and EOCs should be considered at the subnational level in case of emergency de-escalation actions.

*Pillar 2, Risk Communication and Community Engagement (RCCE),* the COVID-19 SPRP *emphasized* communication and students’ participation through the identified Platforms for risk communication and rumors management. The RCCE coordination mechanism lays out the rationale and actions for integrating communities at the core of the response and connecting proactively^17^. To promote students’ adherence to the SOPs, transforming complicated scientific knowledge required clear and actionable information based on community perceptions through the identified platforms and regular campaigns^17^.

*Pillar 3, Surveillance, case investigation, and contact tracing*. The argument over the several transmission routes and scenarios for COVID-19 spreading was going on^32,33^. At the subnational level, HEIs may face more than one of these transmission scenarios^14^. The WHO suggests using the Transmission Scenarios Classification during the formulation of the COVID-19 SPRP and for the implementation of PHSM^19,27^. According to WHO guidance on global surveillance for COVID-19, the university began with scenario one in the first week of the exam and ended with scenario three by the end of the exams. Three positive cases were detected in one faculty, all clustered in time and geographic location^19^. This may be attributed to the study nature and the direct communication with patients in this faculty. In June 2021, the university utilized Go. Data software as an outbreak investigation tool aids in pinpointing the source of the outbreak and visualizing the transmission chain between students^34^.

*COVID-19 Rapid Response Team*, the key principle behind the multidisciplinary Rapid Response Team is to be trained in public health emergencies and respond rapidly in a coordinated manner^22^. Early intervention and leadership commitments can reduce morbidity and mortality^35^ RRT implementation in universities was limited by the faculties' multiculturalism, and traditional hierarchical order models even of rapid response, this was also agreed on by several studies^36^.

*Pillar 4, Points of entry, international travel and transport, and mass gatherings*. One of the significant challenges was screening at the POEs. The use of advanced screening methods with respect to the PHSM on the university gates and in transportation was recommended, as was the announcement of Emergency Contingency Plans^7^. To avoid overcrowding in the POEs, one gate was selected for each faculty. Sorting the gates according to the added value time by using VSM reduced the non-value-added time and improved the process cycle efficiency^24^.

The SOPs' process mapping and flow charts at the POEs were aligned with university IMS and EOCs^23^. It was proved the efficacy of using process mapping as a tool during the outbreaks, to aid in a proactive and prompt response. Students, logisticians, RCCE, IPC experts, laboratory specialists, and incident managers could all familiarize themselves with this process^23^.

*Pillar 5, Adherence to IPC measures was implemented in the SOPs*. The objectives of the PPE plans are to ensure compliance with the PHSM and to provide access to enough PPE in coordination with logistics^6^. This was achieved by sufficient supply management, determining IPC surge capacity requirements, and developing strategies for the appropriate use of PPE in all university settings^6^.

*Pillar 6, National laboratory system* Due to global shortages of laboratory supplies, most countries' testing capacity was insufficient. And so, Rapid Antigen Diagnostic Tests (RADTs) were recommended as screening tools following assessments^26^. Even while RADT is less sensitive than RT-PCR, it can be used in the early detection of infections^37^. On the other hand, implementing RADT for all students proved to be a big logistical challenge, rendering it unsuitable for larger events.

*Pillar 7, Case management and knowledge sharing,* the referral policy here emphasized the necessity of information-sharing between MOH and the private sector on their capacity for critical COVID-19 patients to assure continuity of service. Available bed capacity and occupancy are organized through the integrated hospital referral system to overcome the oxygen delivery shortage and lack of equipment in intensive care units.

*Pillar 8, Operational support and logistics in the management of supply chains and the workforce* the importance of facilities needs assessment, logistics, and surge capacity assessments were to assess the workload capacities of the university buildings and assure high levels of social distancing^38^. According to WHO guidelines for COVID-19 risk communication packages, ventilation is one of the most important IPC measures^28^. German study showed that three hygiene concepts in indoor events, with IPCs measures and adequate ventilation, reduce the spread of SARS-CoV-2^39^. The facilities assessment revealed that each building required EMS medical support services. The aim of identifying private exits in each building for EMS was to prevent the transmission of COVID-19 among facilities^18^.

The main scope of *Event Medical Services* was assessing vital signs, treating onsite minor injuries, and providing triage. Even during the pandemic, the EMSs established as mobilized surge capacity for supporting screening establishing rapid access and eliminating the busy POEs. The operational guidelines of the event medical services EMS emphasized the IPC measures, ventilation rate, triage process mapping, special transportation of suspected cases, and the process of removing biohazard and non-biohazard waste^18^.

Successful administration of the Essential Supplies was aided by supply chain and logistics regulations. An inadequate supply of PPEs was avoided by using an accurate forecasting tool^29,40^. The unexpected increase in the need for PPE caused by the COVID-19 pandemic has caused PPE shortages, posing a tremendous challenge to the PPE supply chain, and coordination with the partners to accommodate the needs in accordance with the national Roadmap for Improving PPE Production, procurement, and emergency logistics^41^.

*Pillar 9, Maintaining essential health services during the COVID-19 outbreak,* COVID-19 SPRP aims to build resilient health systems capable of ensuring the continuity of services during emergencies considering the physical and mental well-being of the workforce. Furthermore, a system for monitoring the provision of these services is required.

*Pillar 10, other possible topics,* about 50 of 300 COVID-19- database specific trigger questions encompass the students with disabilities, gender equality, equity, and human rights (Country COVID-19 intra-action review (IAR): trigger question database)^12^. Higher Education Providers must readjust the education policies and procedures for responding to the COVID-19 emergency to accommodate the disability perspective, as clearly reflected in the United Nations Convention on the Rights of Persons with Disabilities. We must consider the needs of disability, gender equality, and disadvantaged population in the formulation of education policy in all the emergency preparedness and responses in the future^9^.

### Theoretical contribution

Countries continue to deal with various COVID-19 transmission scenarios, and so the WHO emphasizes the necessity of conducting intra-action reviews^13^.

The operational management of WHO's ten pillars can be customized to match the national or subnational and, organizational context^6^. The reporting templates of the IAR can be used to assess the COVID-19 operational planning guidelines^12^.

Through interactive sharing and systematic reviews, we were able to meet the IAR objectives of identifying best practices, gaps, and obstacles as well as how to strengthen the COVID-19 response in higher education institutions^42^.

As Higher education providers we conduct system-level root causes through the monitoring and evaluation framework, imply accurate steps for constructing a resilient higher education system, and institutionalize best practices for long-term improvement of the ongoing rapid response^7^.

The most compelling IAR evidence is the significance of synchronization between the ten pillars in preventing COVID-19 transmission. The gaps in PPE supply chain management, for example, will impair infection prevention control measures, causing virus dissemination in case management and will interrupt essential services during the outbreaks, disrupting contact tracing capabilities, and increasing info emic and rumors in the community.

This IAR has contributed to the improvement of the COVID-19 response in the university by initiating a systematic response, defining strategic response pillars, and incorporating multiple sectors as a whole-of-society response to approach the pandemic^12^. Some improvements have been evidenced since the implementation of IAR recommendations in April 2021, for example, regular assessments of the COVID-19 response plan KPIs have reinforced stakeholders’ coordination, and rapid response teams’ legal framework has been activated^7^. GO.DATA is used as contact tracing software^34^. The performance of all participants improved, as well as community engagement and student compliance with PHMS^27^. As a result, the incidence of new cases decreases in the following semesters.

The findings from IARs can be used to influence decision-making for continual improvement of the operational planning guidelines during the outbreaks on the subnational, national, and regional levels. This finding can be used as input for the WHO IHR (2005) Monitoring and Evaluation Framework and review of the National Action Plan for long-term and high levels of Readiness–Capacity.

## Conclusion

The results of this study suggested that the harmonization in the ten-pillar performance and the high level of Readiness–Capacity during mass gathering events contributed to reduced COVID-19 transmission between students during on-campus exams. The COVID-19 pandemic has yet to reach its final stages, and so there are still many areas that could be improved. These areas require immediate action continuous monitoring and multisectoral coordination to ensure immediate and rapid responses through later waves of the pandemic. Future pandemic waves could overwhelm the higher education system and healthcare infrastructure if proactive planning is not appropriate.

## Supplementary Information

Supplementary Information 1.

Supplementary Information 2.

Supplementary Information 3.

Supplementary Information 4.

Supplementary Information 5.

## Data Availability

The datasets generated and analyzed during the current study are available as Supplementary files.
