# Supplementary material for: Assessment of COVID-19 preparedness response plan on higher education students simulation of WHO intra-action review in Egypt
Source: Sci Rep. 2023 Jan 13;13:741. doi: 10.1038/s41598-023-27713-1 (PMC9839230; doi:10.1038/s41598-023-27713-1)
Supplement: Supplementary file 1 — Supplementary Information 1. [file 41598_2023_27713_MOESM1_ESM.pdf]

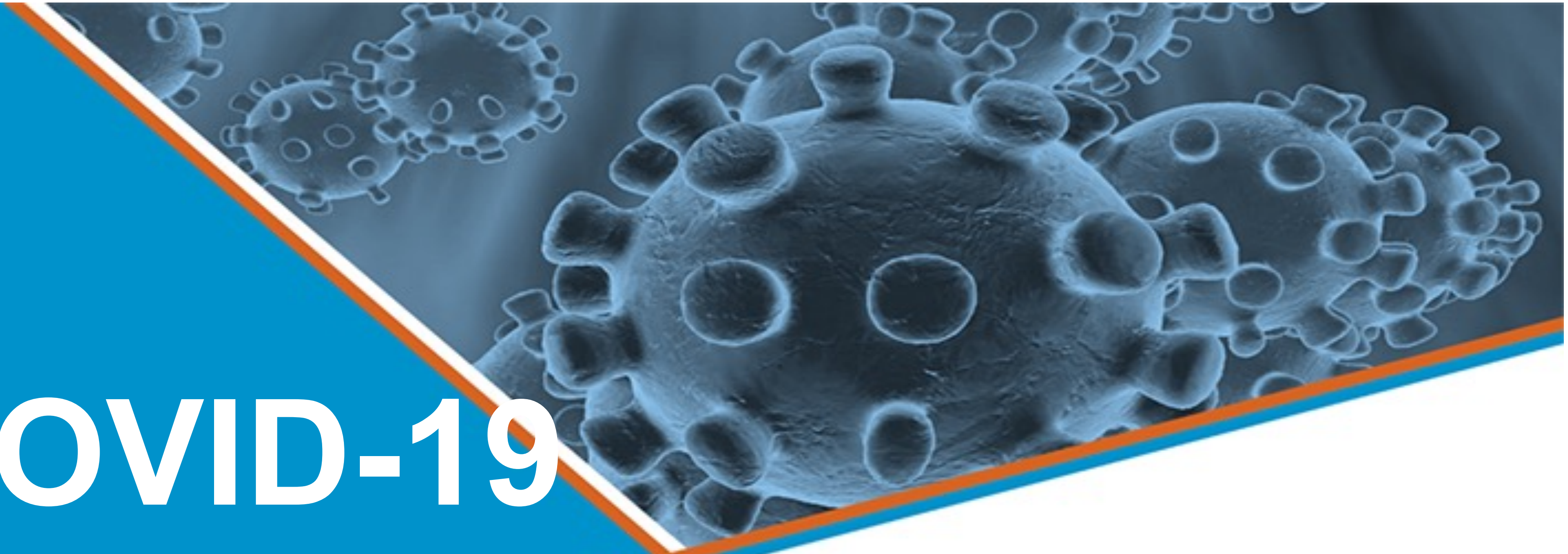

COVID-19

O6U INTRA-ACTION REVIEW (IAR)

جامعة ٦ أكتوبر
OCTOBER 6 UNIVERSITY

EGYPT
Jan_2021

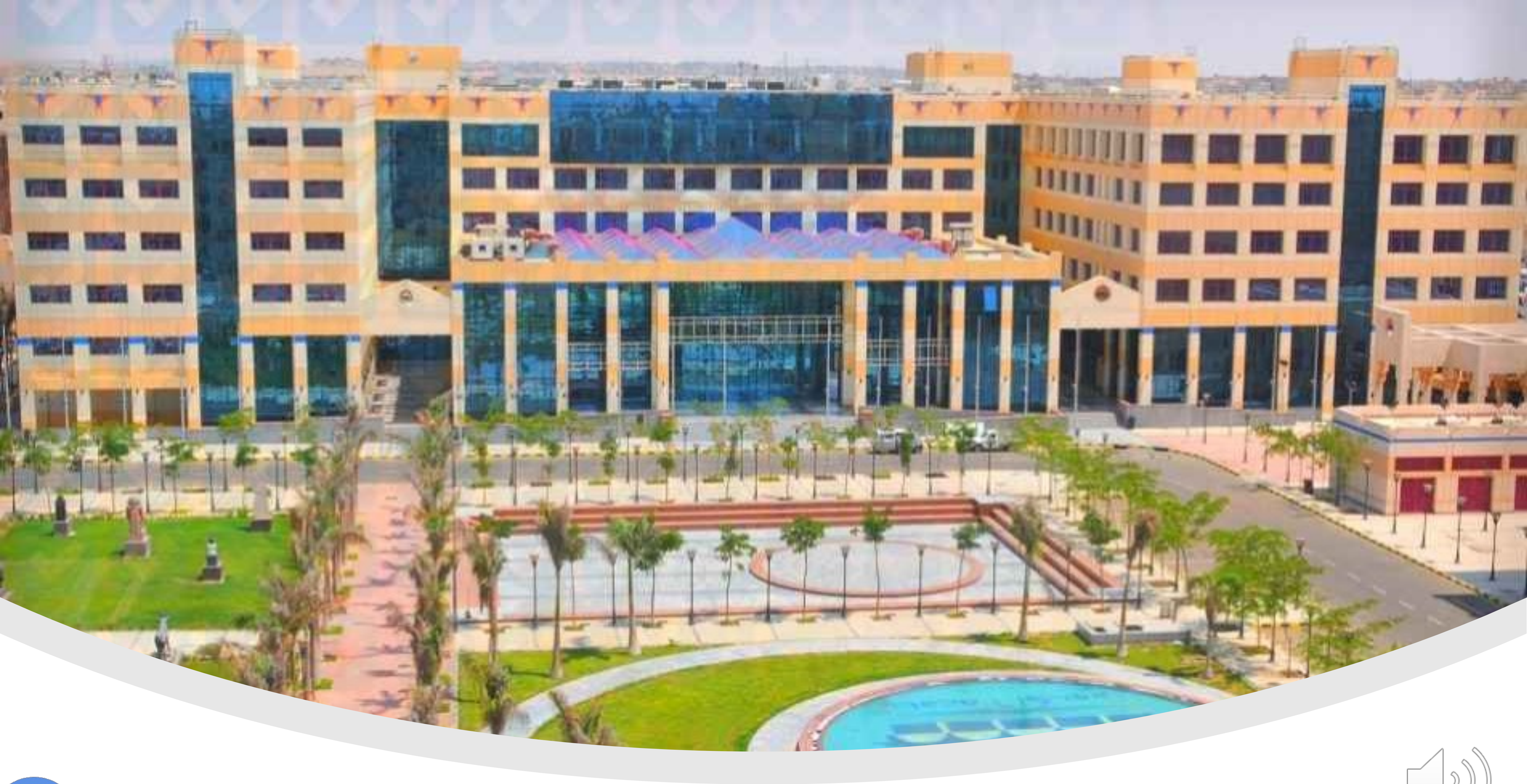

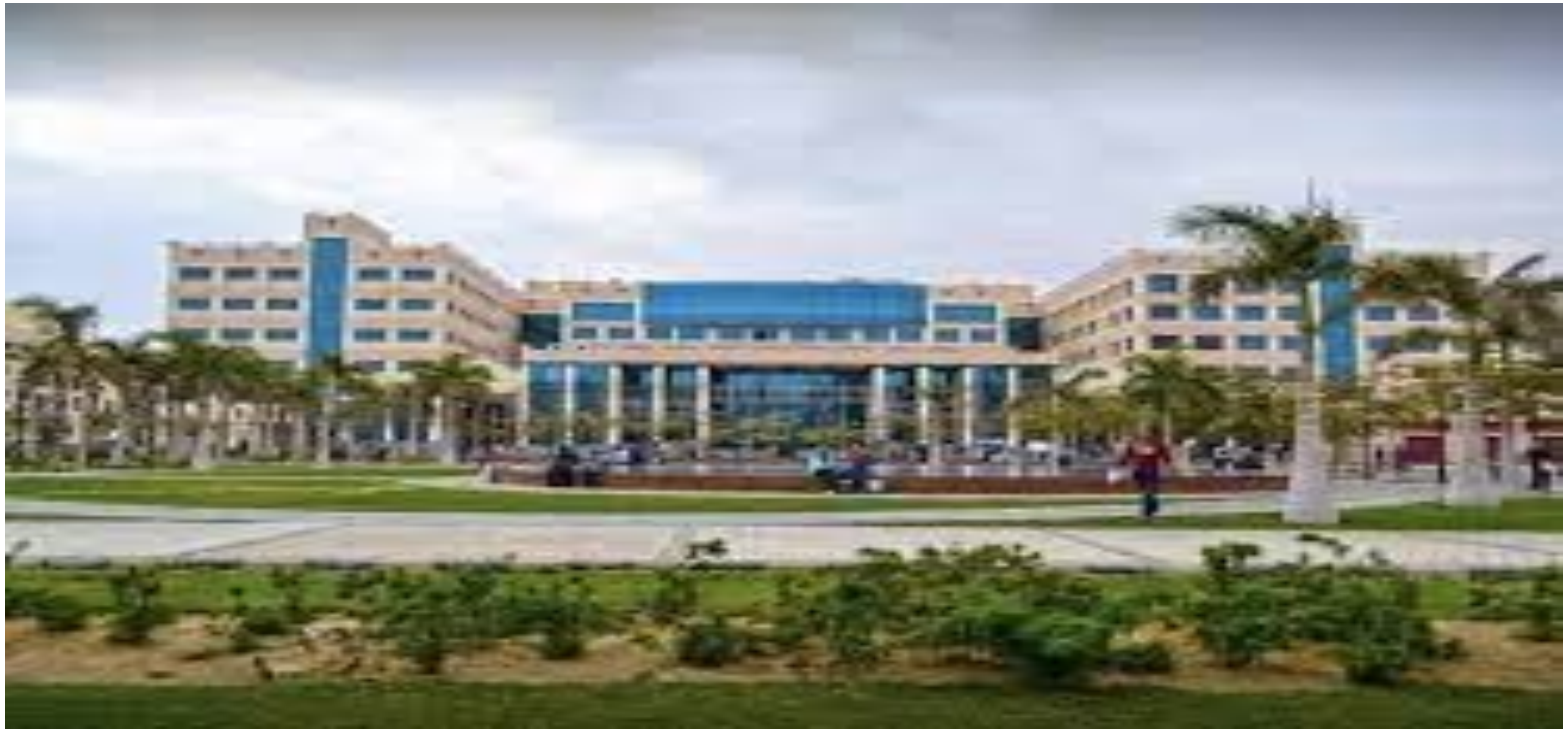

Professor.Dr Gamal Samy

The president of O6U

IAR Index

- Introduction
- Planning phase (Pre_IAR)
- Operational phase (During _IAR))
- Presenting IAR results and follow-up actions
- Post-event phase (AAR)

IAR_ Introduction

جامعة ٦ أكتوبر
OCTOBER 6 UNIVERSITY

Adapted from After Action Review guidance

"… implement in-depth reviews of significant disease outbreaks and PH events. (IHR Review Committee Recommendations - Resolution WHA68.5 in May 2015)

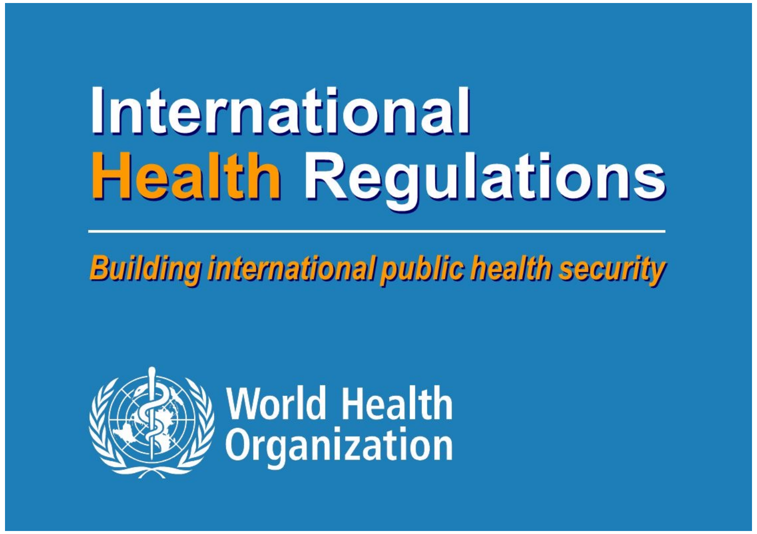

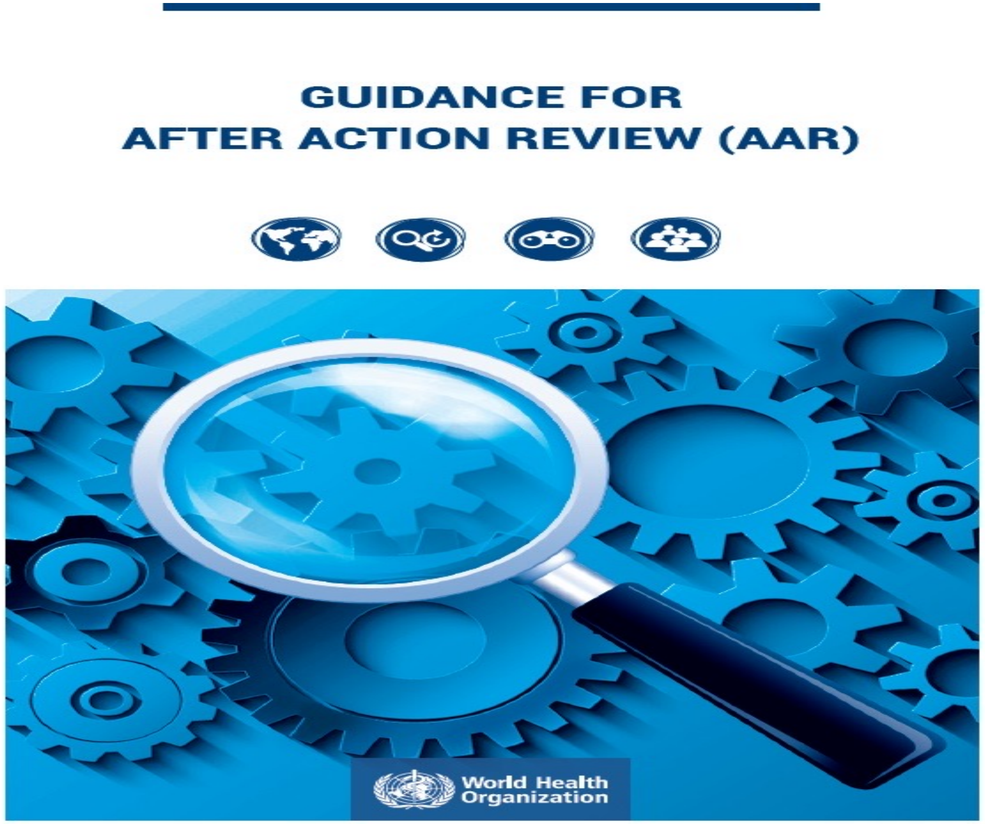

جامعة ٦ أكتوبر
OCTOBER 6 UNIVERSITY

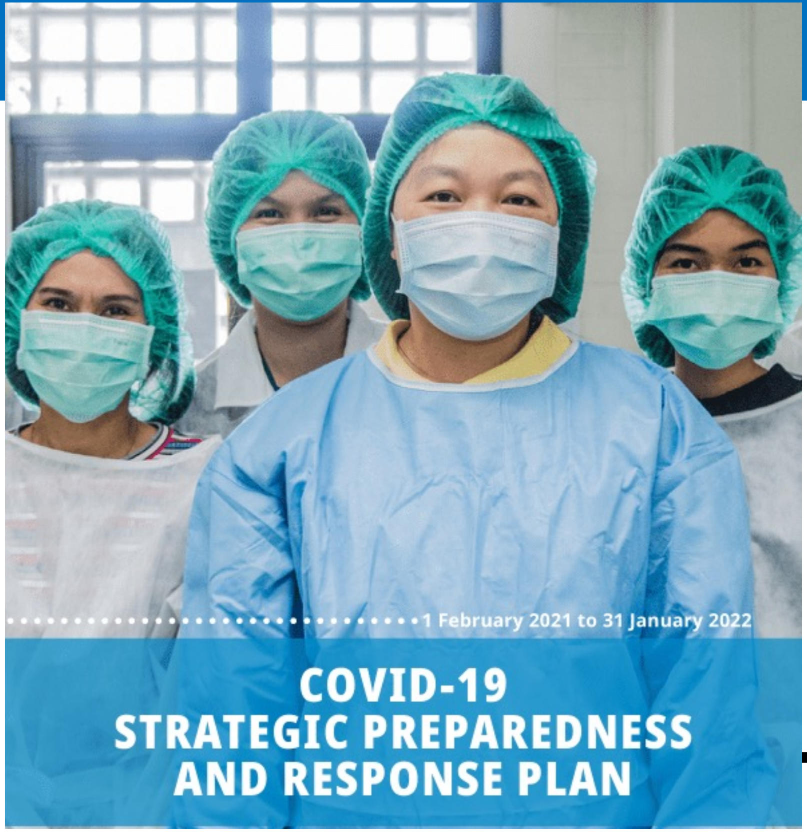

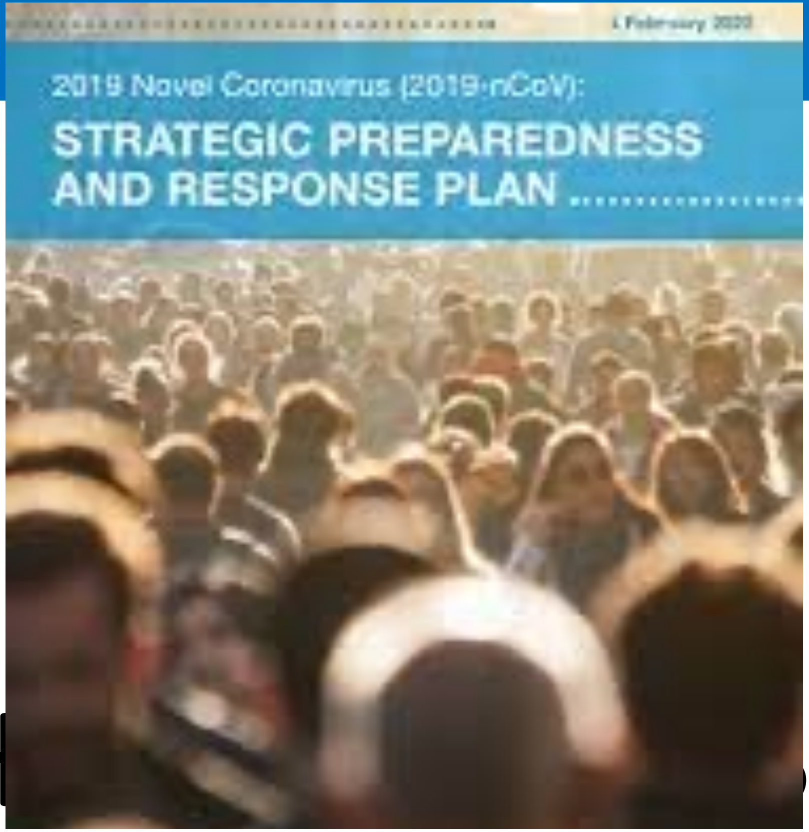

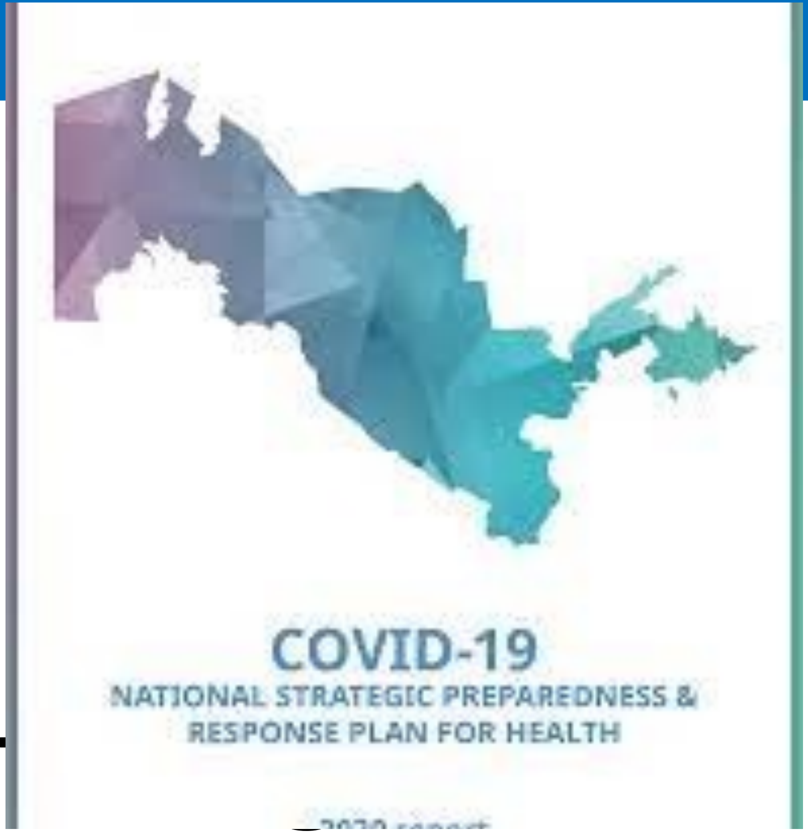

Preparedness and response plan (SPRP) 2020-2021

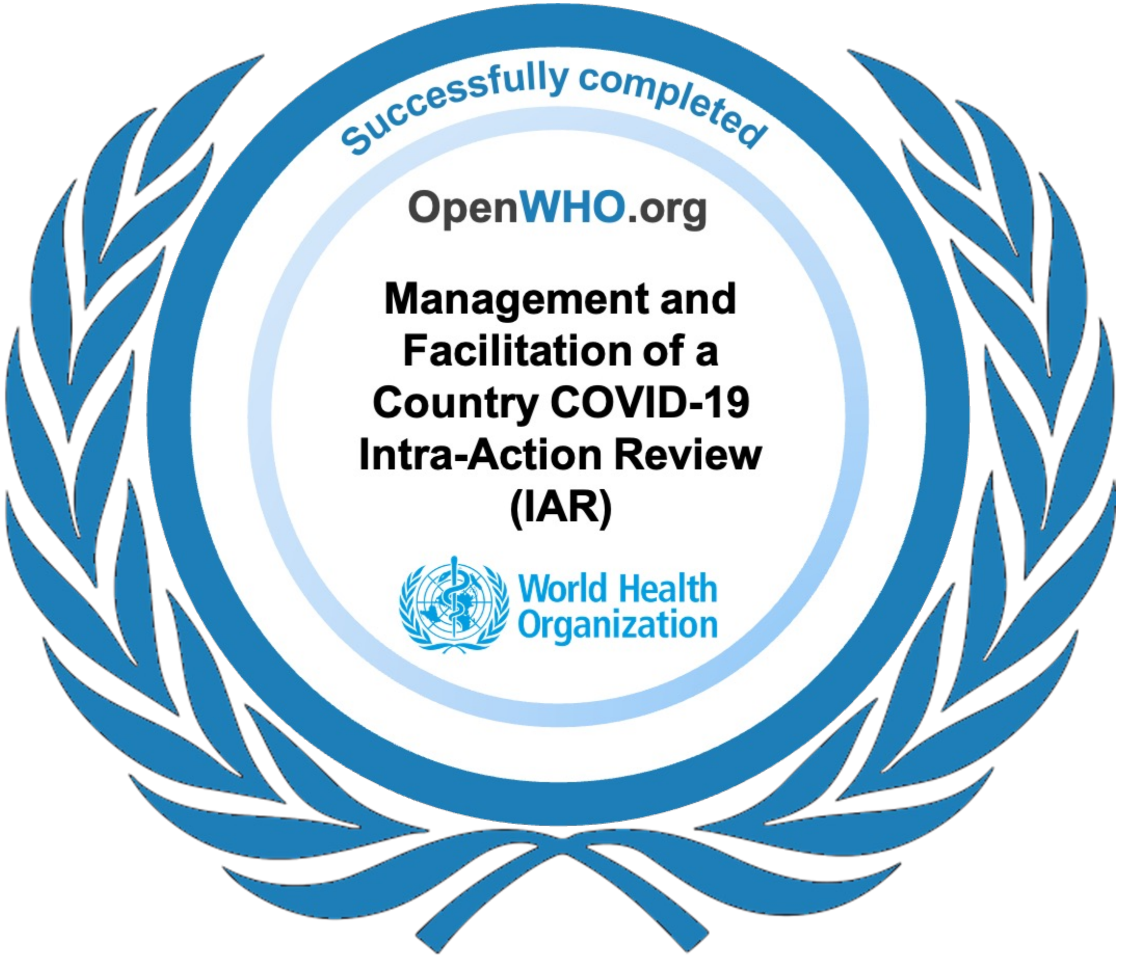

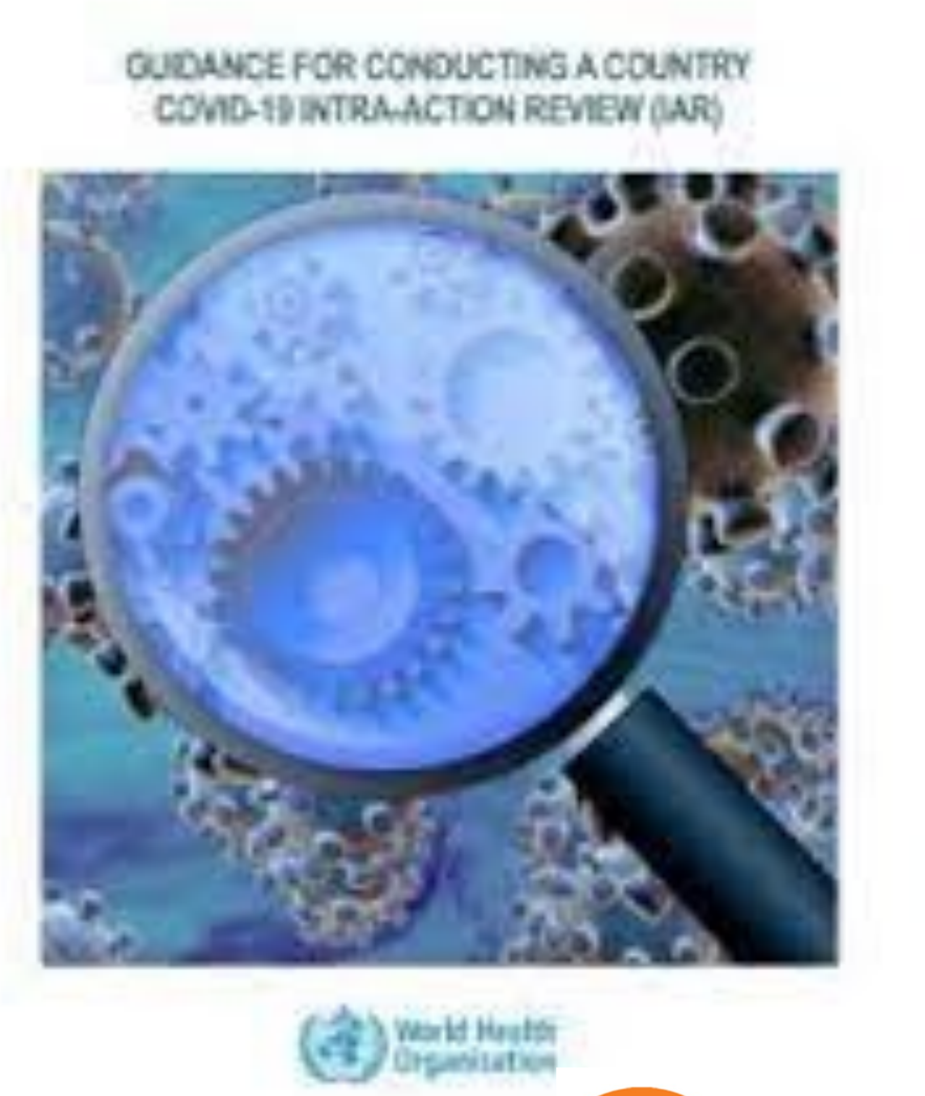

جامعة ٦ اكتوبر
OCTOBER 6 UNIVERSITY

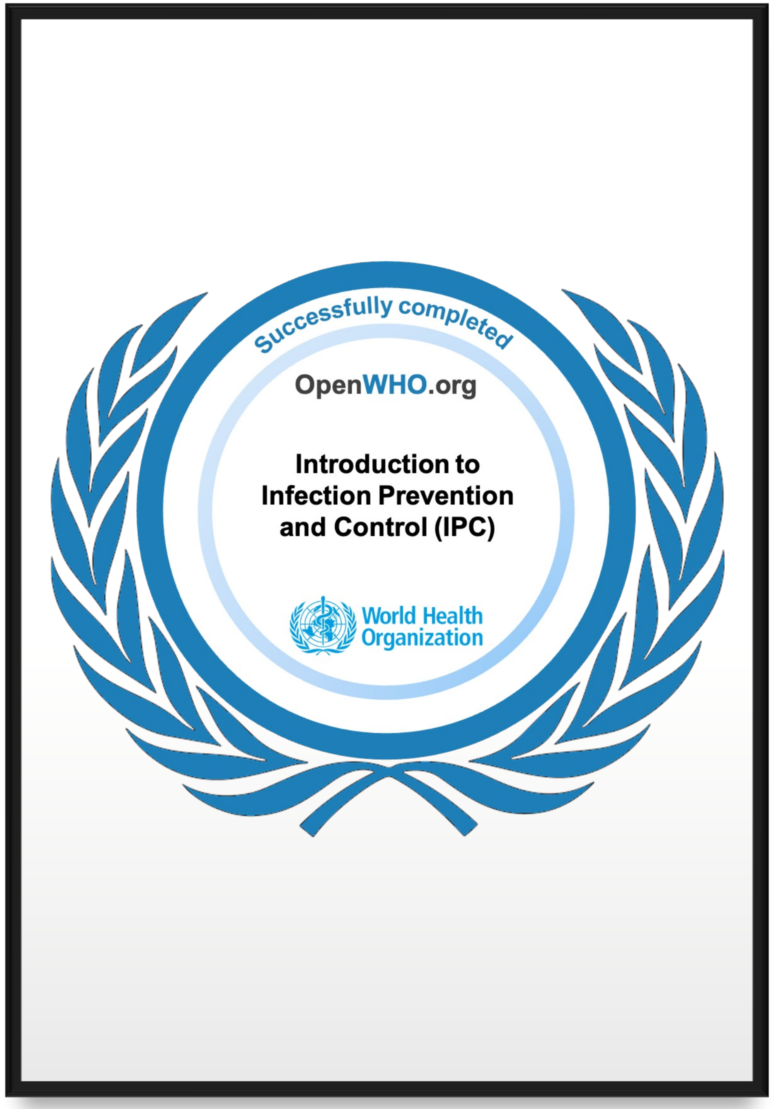

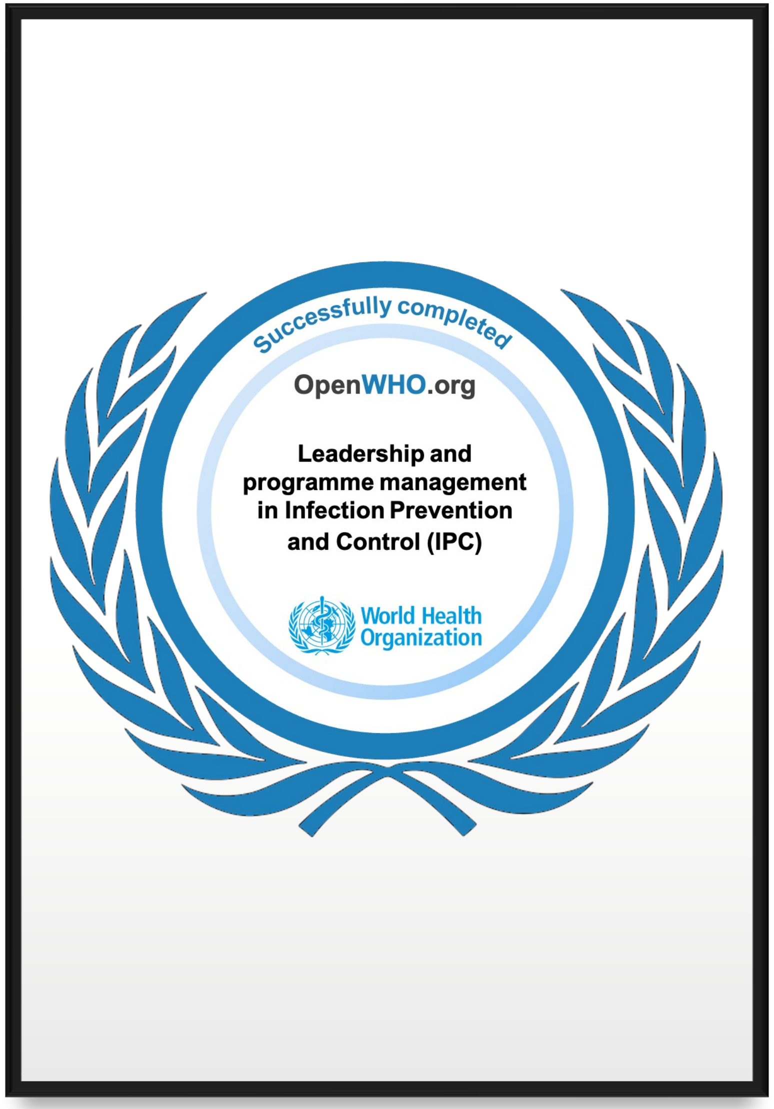

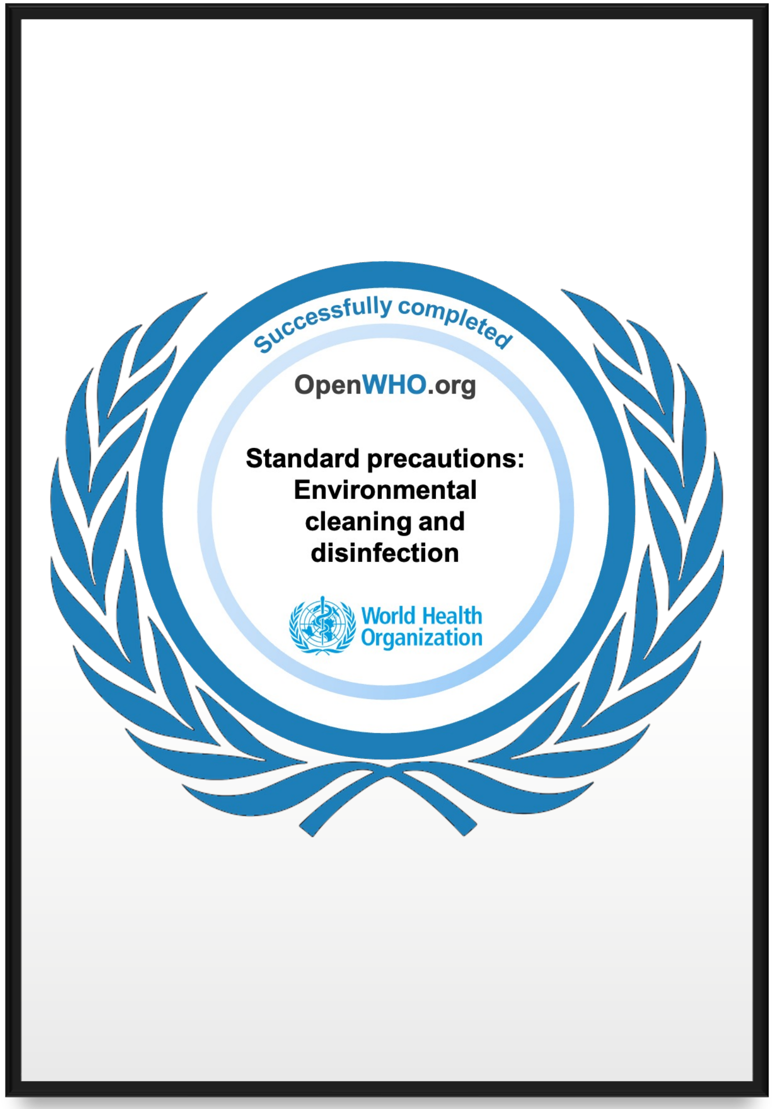

جامعة ٦ أكتوبر
OCTOBER 6 UNIVERSITY

WHAT IS AN INTRA-ACTION REVIEW?

An Intra-Action Review is

a **qualitative review** of

actions undertaken so far

to **respond** to an ongoing **emergency**

as a **means of identifying**

gaps, **lessons** and **best practices**

in order to **improve the response plan.**

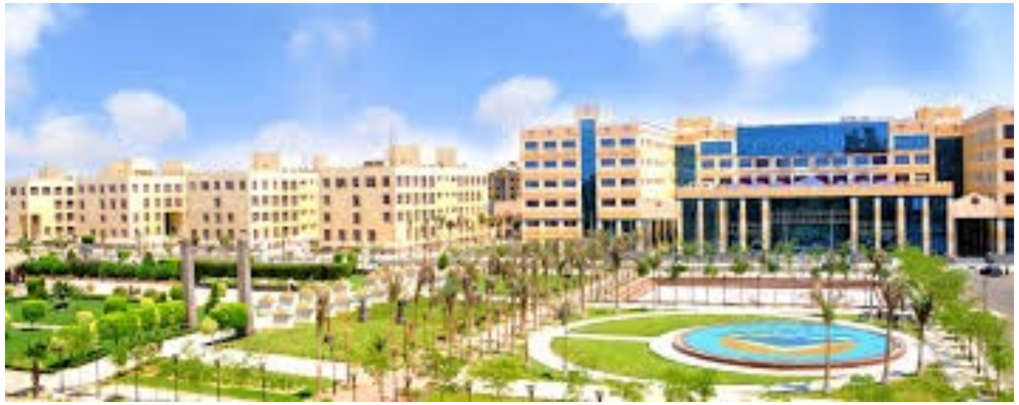

#	Pillar	Update
1	Country-level coordination, planning and monitoring	Additional questions
2	Risk communication, community engagement, and infodemic management	Additional questions
3	Surveillance, case investigation and contact tracing	Additional questions
4	Points of entry	Additional questions
5	National laboratory system	Additional questions
6	Infection prevention and control	Additional questions
7	Case management and knowledge sharing about innovations and the latest research	Additional questions
8	Operational support and logistics in the management of supply chains and workforce resilience	Additional questions
9	Strengthening essential health services during the COVID-19 outbreak	Additional questions
10	COVID-19 vaccination	**New pillar**
11	Vulnerable and marginalized populations	**New pillar**
12	National legislation and financing	**New pillar**
13	Public health and social measures	**New pillar**
14	Other possible topics and cross-cutting issues	Additional questions

WHAT IS AN INTRA-ACTION REVIEW?

A good practice for continuous **collective learning**

by bringing together

the **relevant stakeholders**

to **critically** and **systematically**

analyse actions undertaken so far in the response

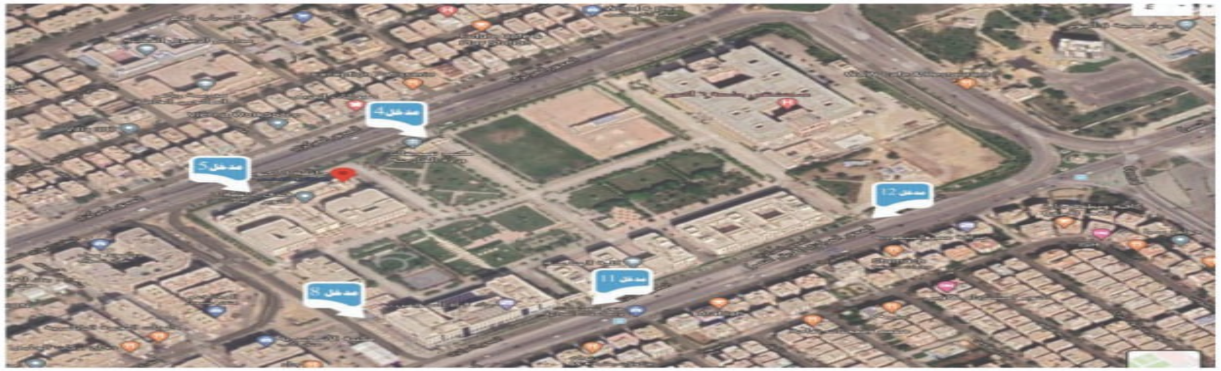

WHAT IS AN INTRA-ACTION REVIEW?

IAR are a **constructive**, **collective learning** opportunity,

where **stakeholders** of an emergency response **within** the health sector or **between** sectors,

can find **common ground** on **how to improve** preparedness and response **capability** for the current emergency

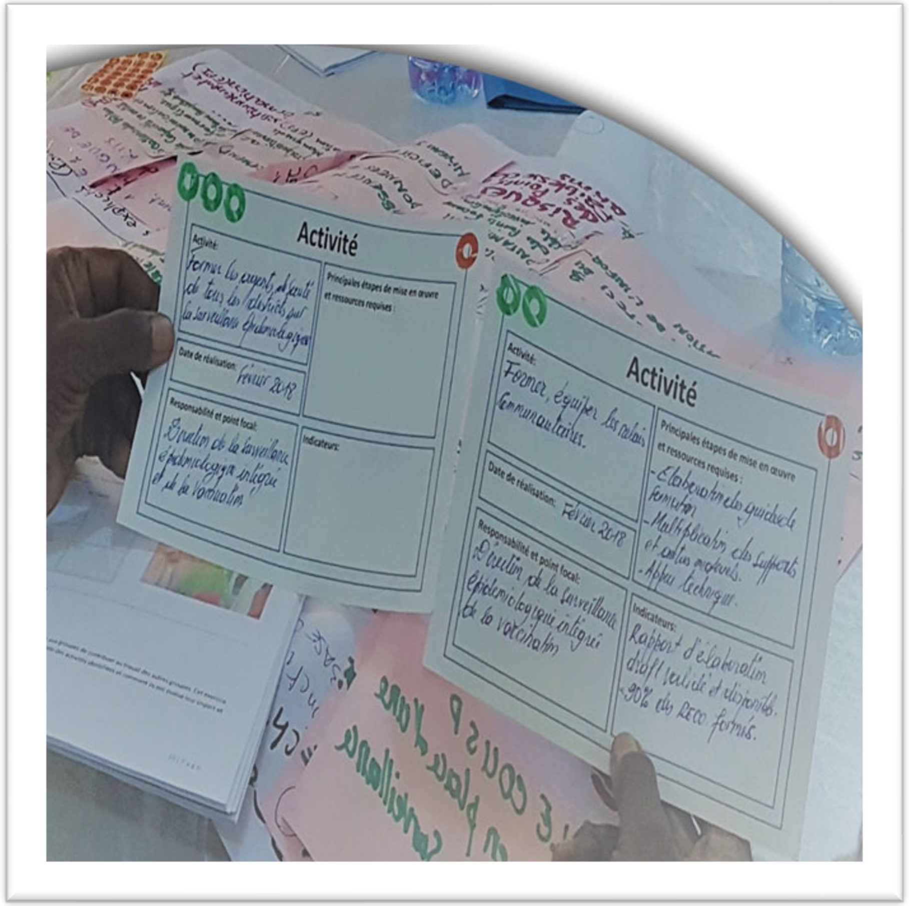

Principles

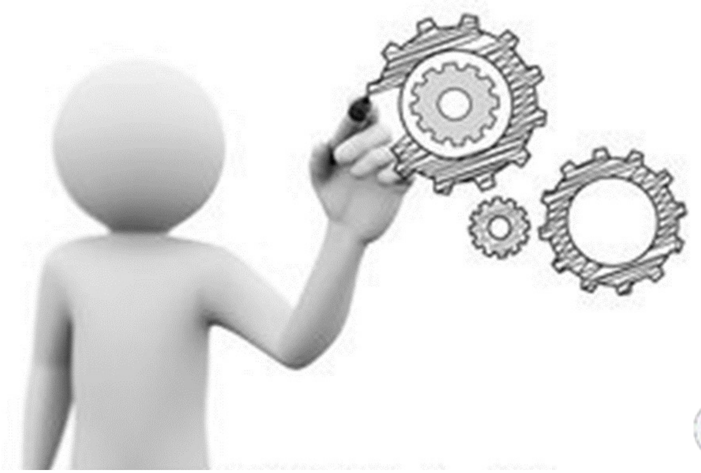

- Participative
- Open and honest spirit
- Space for experience sharing and mutual learning
- Analysis of systems and processes
- Oriented towards the identification of solutions
- A compilation of participants' perceptions

What an Intra-Action Review is not?

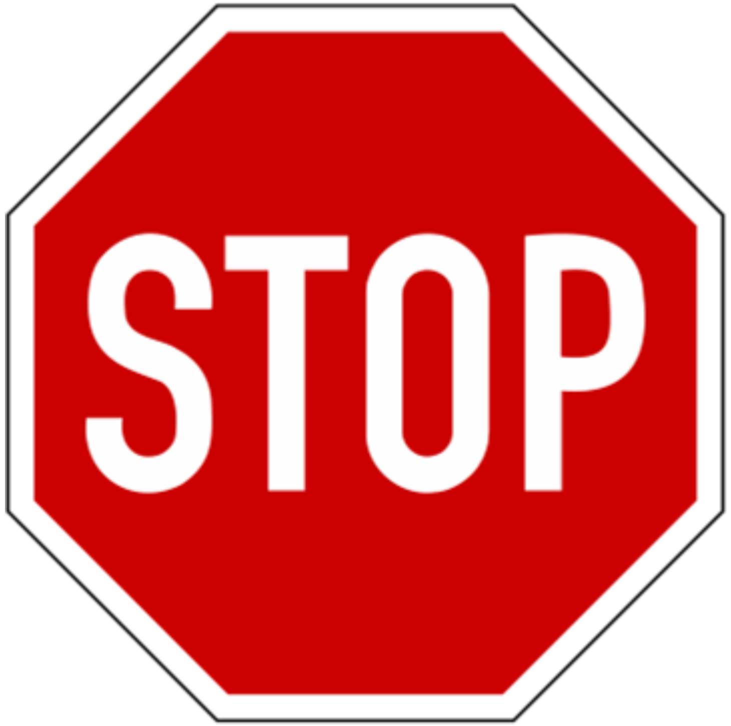

Intra-Action Reviews are **not**:

- an external evaluation of an individual's or a team's performance;

- an opportunity to criticize, blame or judge individuals.

Intra-Action Reviews do not measure performance against benchmarks or key performance standards.

KEY PHASES CARRIED OUT DURING THE REVIEW

OBJECTIVE OBSERVATION

Establish how actions were actually implemented during the response, in contrast to how they are supposed to or usually happen, according to plans and procedures.

ANALYSIS OF GAPS & BEST PRACTICES AND CONTRIBUTING FACTORS

Identify the gap between planning and practice.

Analyse what worked well and what worked less well and why.

IDENTIFICATION OF AREAS OF IMPROVEMENT

Identify actions to strengthen or improve performance
and how to follow-up.

IAR_ Planning phase (Pre_IAR)

INTRA-ACTION REVIEW OVERVIEW

Introduction: **Response plan and actual timeline of the response**

STEP 1

Step 1: **What went well? What went less well? Why?**

STEP 2

Step 2: **What can we do to improve the COVID-19 response?**

STEP 3

Step 3:**The Way Forward**

Objectives

The objectives of the IAR are to identify best practices, restricting variables, and challenges, strengths and gaps in the university's COVID-19 response on the sub-national level and document all of these using WHO IAR notetaking templates for long-term actions.

Scope of the COVID-19 Intra-Action Review

The IAR roadmap process is divided into three phases, (pre, during, and post). The pre-IAR phase [13], started one week before the exams. We used the WHO Mass Gathering COVID-19 Risk Assessment Tool–Generic Events.

The primary outcome was the number of positive SARS-CoV-2 PCR tests during the exams till one-week post-exam gathering from the illegible students. The secondary outcome was to assess the efficacy of the Strategic Preparedness and Response Plan on preventing COVID-19 transmission during on-campus exams regularly using Readiness–Capacity KPIs. The anticipated cut-off of the KPIs score (is 85%-80%).

Scope of the COVID-19 Intra-Action Review

Properly identify Risk communication and community engagement methods.

Ensure Surveillance, case investigation and contact tracing.

Identify the Points of entry.

Monitoring of Infection prevention and control measures application.

Determine the methods of Case management system.

Operational support and logistics in the management of supply chains and the workforce

Maintaining essential health services during the COVID-19 outbreak

INTRA-ACTION REVIEW OVERVIEW

Introduction: **Response plan and actual timeline of the response**

STEP 1

Step 1: **What went well? What went less well? Why?**

STEP 2

Step 2: **What can we do to improve the COVID-19 response?**

STEP 3

Step 3: **The Way Forward**

Operational phase (During _IAR))

Implementation phase

During _AIR

جامعة ٦ أكتوبر
OCTOBER 6 UNIVERSITY

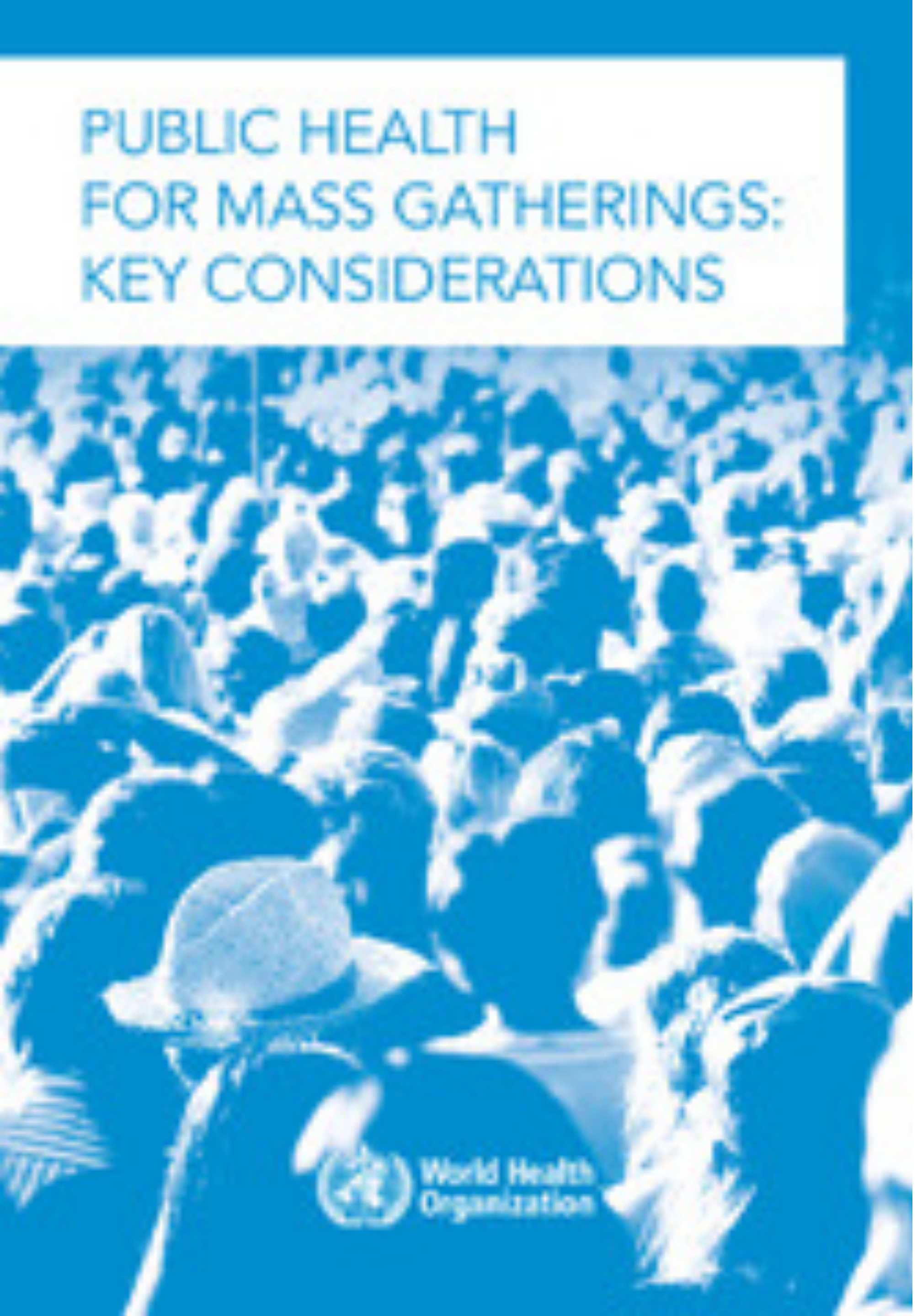

Public health for mass gatherings: key considerations

Citation

World Health Organization. (2015). Public health for mass gatherings: key considerations. World Health Organization. https://apps.who.int/iris/handle/10665/162109

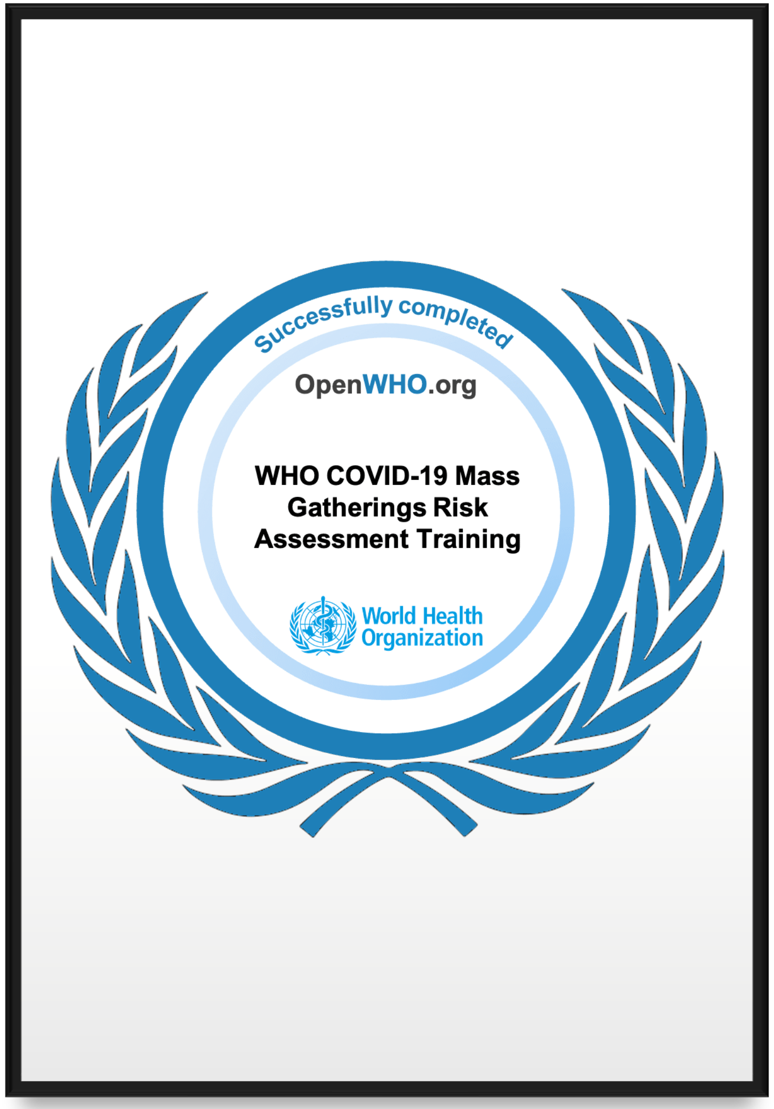

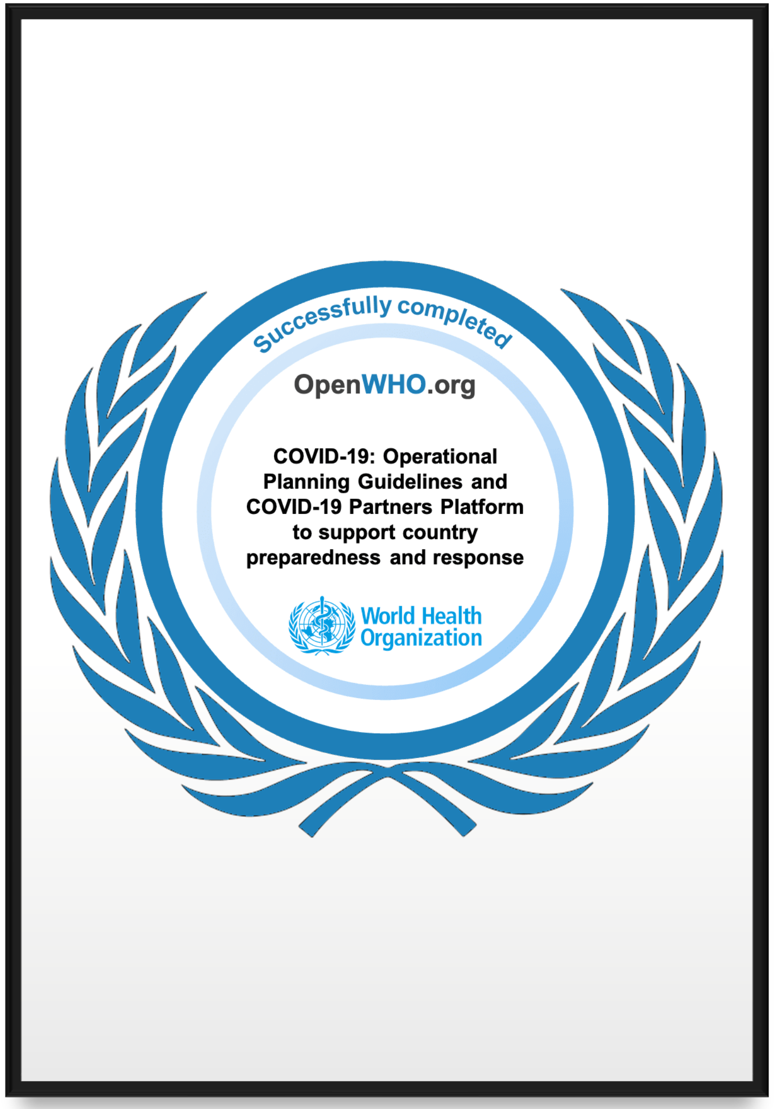

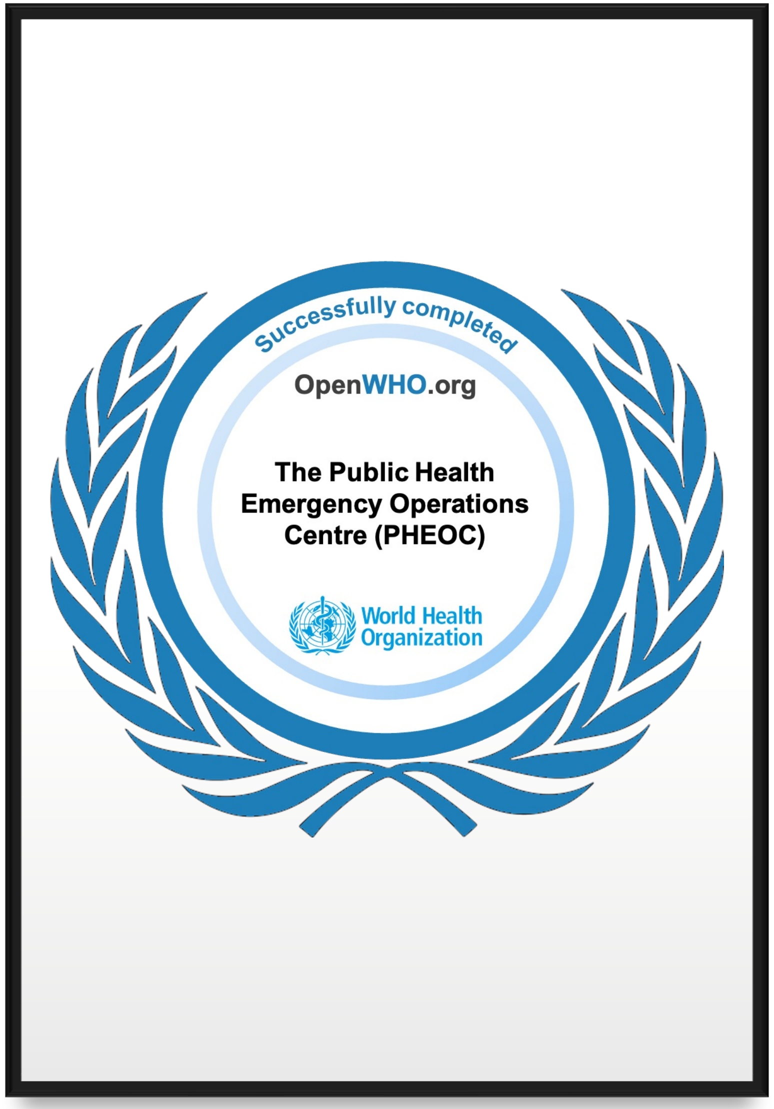

جامعة ٦ أكتوبر
OCTOBER 6 UNIVERSITY

WHO Mass Gathering COVID-19 Risk Assessment Tool

Total Risk Assessment Score from COVID-19 Risk Evaluation Tab	1
Total Mitigation Score from COVID-19 Mitigation Tab (%)	79

Risk Versus Mitigation Decision Matrix				
	Total Mitigation Score (%)			
Total Risk Score	76-100	51-75	26-50	0-25
0	VERY LOW	VERY LOW	VERY LOW	LOW
1	VERY LOW	LOW	LOW	MODERATE
2	LOW	LOW	MODERATE	MODERATE
3	MODERATE	MODERATE	HIGH	HIGH
4	HIGH	HIGH	VERY HIGH	VERY HIGH
5	VERY HIGH	VERY HIGH	VERY HIGH	VERY HIGH

KEY	
VERY LOW	Overall risk of transmission and further spread of COVID-19 is considered <u>VERY LOW</u>

جامعة ٦ أكتوبر
OCTOBER 6 UNIVERSITY

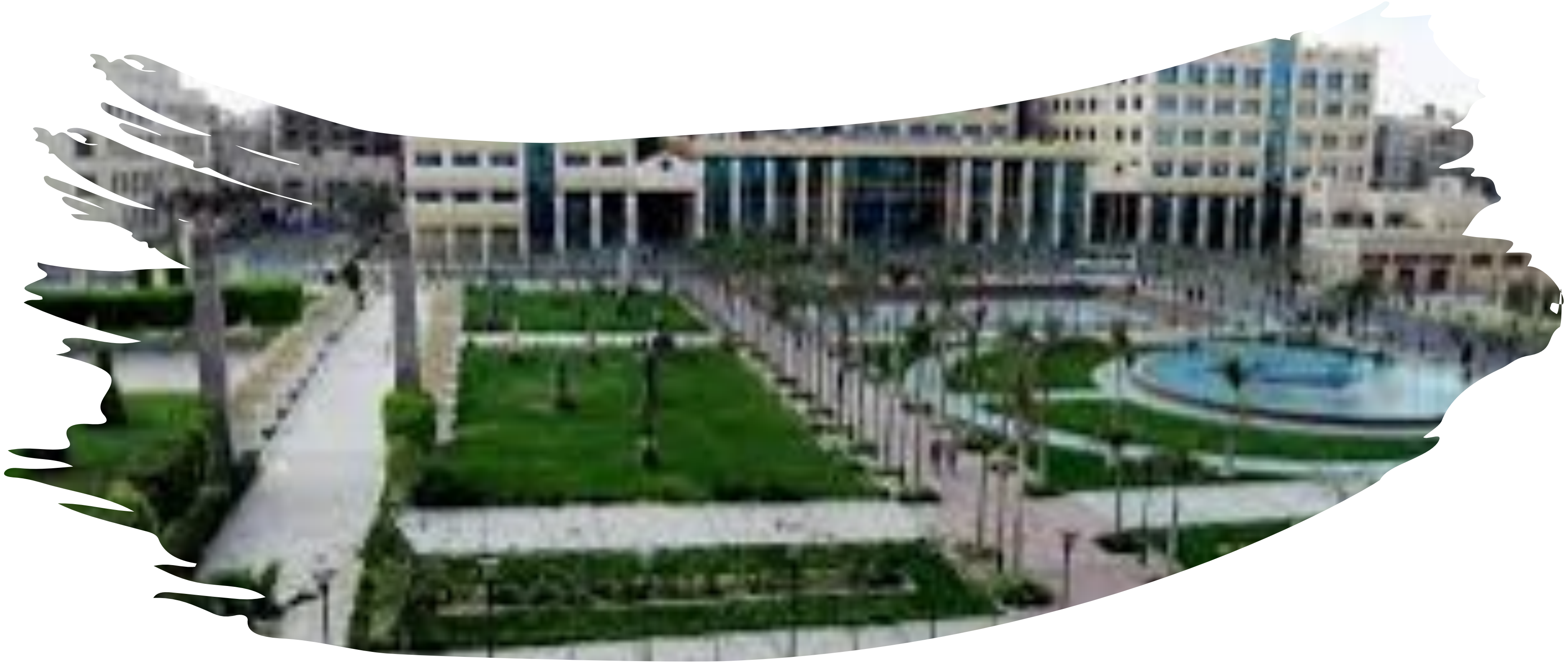

October 6 university COVID-19 Strategic Preparedness and Response Plan

Process mapping Detailed flow diagram – Generic Events

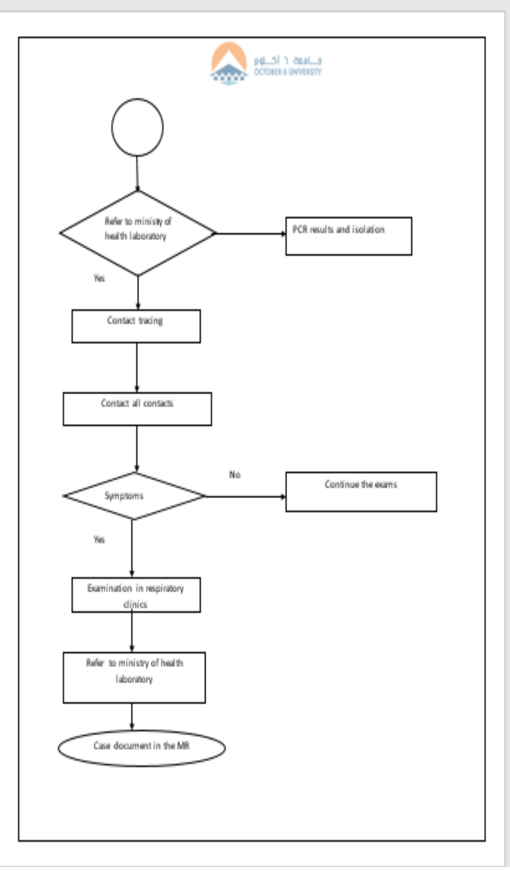

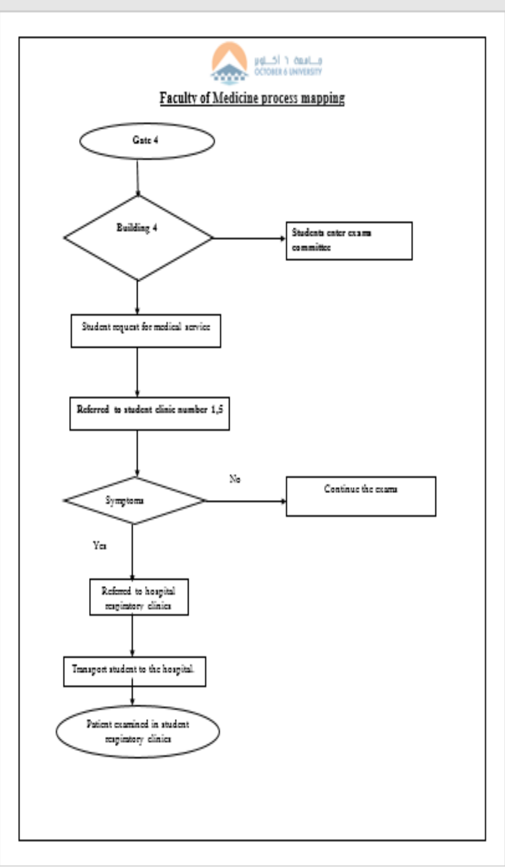

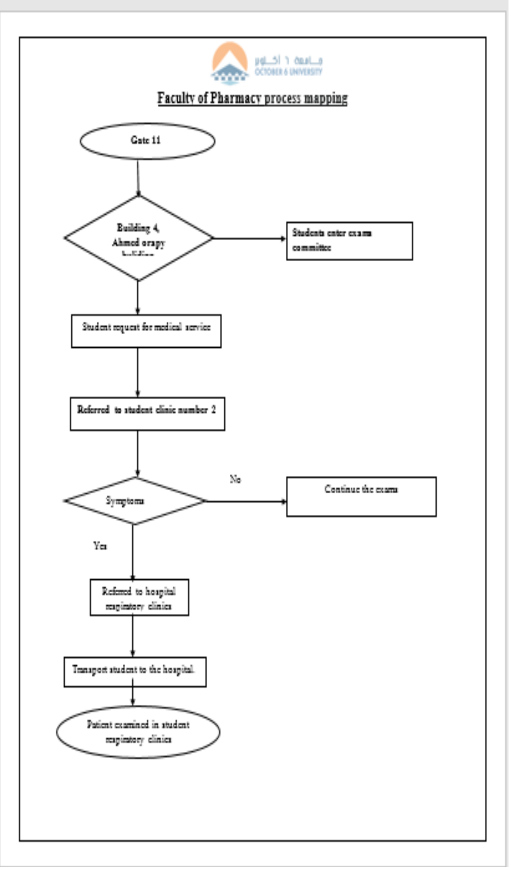

Value stream mapping

Value stream mapping before and after improvement

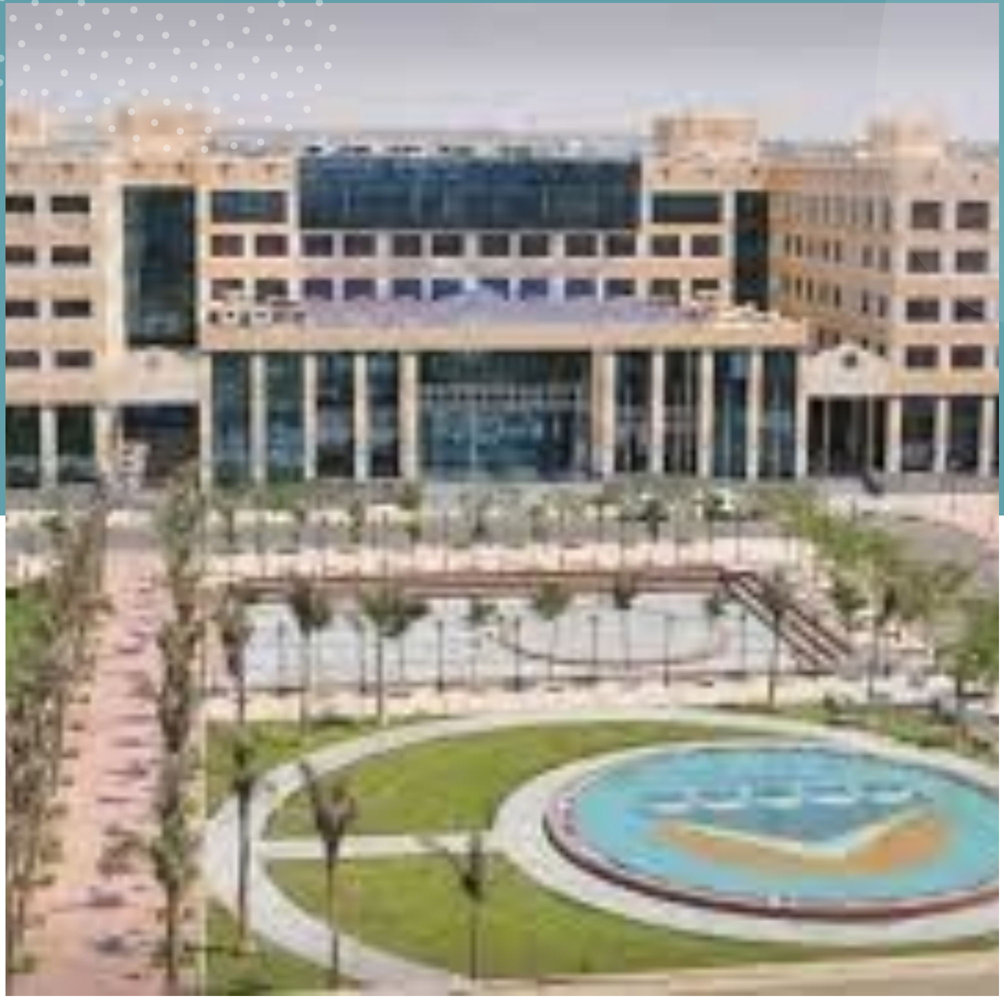

Value stream mapping before the improvement:

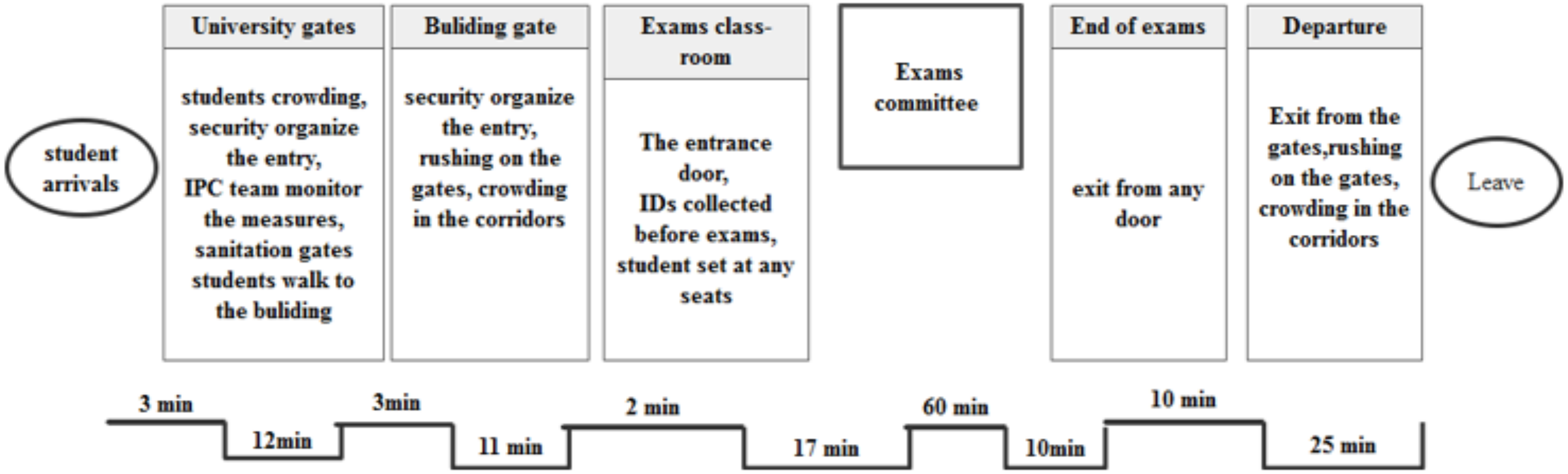

Added value time	78 min
Non-added value time	75 min
Cycle time	78 min
Lead time	153 min

Final percent:

NV/LT percent= 49%

VA/LT percent= 50.9%

Value stream mapping after the improvement:

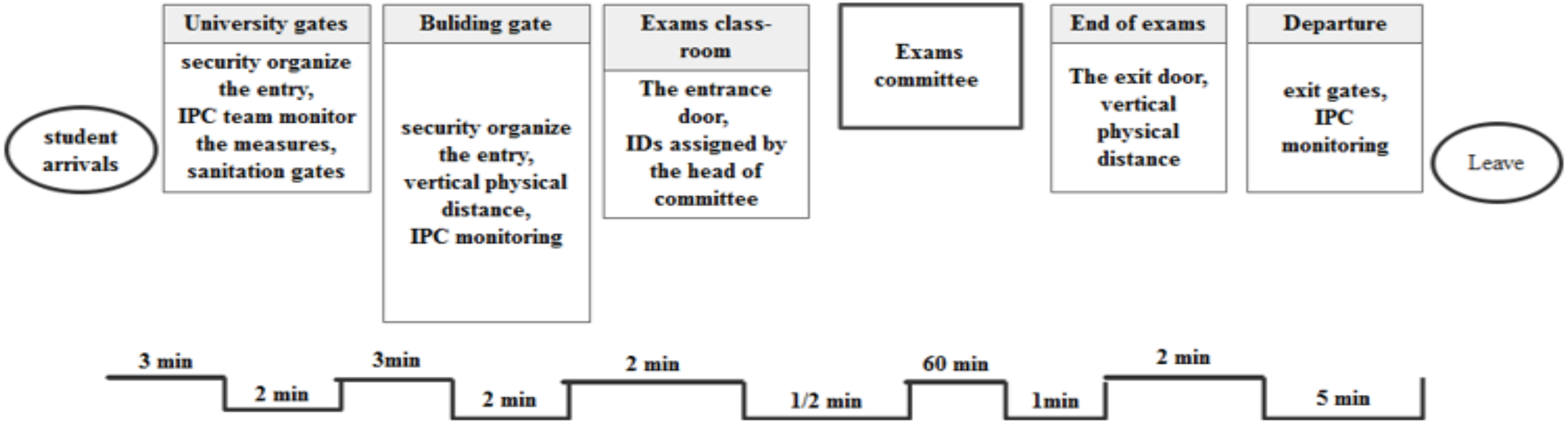

Added value time	70 min
Non-added value time	7 min
Cycle time	70 min
Lead time	77 min

<u>**Final percent:**</u>

NV/LT percent= 9 %

VA/LT percent= 90.9%

MONITORING FRAMEWORK

The key performance indicators listed below will be used to globally monitor the implementation of the 2019-nCoV strategic preparedness and response plan. Systems will be established with national governments and partners to monitor key performance indicators on a regular basis.

Category	Indicator	Target	Rationale for use
Epidemiology situation	Number of countries with cases	N/A	Basic epidemiological data to understand the scale and evaluation of the event. Further analyses and stratification:<br>• Number of confirmed cases worldwide<br>• Number of countries with local transmission<br>• Number of countries with imported cases<br>• % of countries in which there are cases that were not directly associated with travel to areas affected by community spread<br>• % of alerts, suspects or confirmed cases detected at points of entry<br>• % deaths reported among reported cases
	% of cases who are healthcare workers	TBD	This measure can be useful to strengthen IPC over time. Data might be available if online reporting platforms for data sharing are established. Some caution should be added about interpretation as some healthcare workers may have high risk of community transmission.
Global response – Programme management	% Strategic Response Plan (SRP) budget funded	80%	This measure helps to assess the financial support to the global response as per the SRP.
	% of funds received for the SRP implemented	100%	This measure helps to assess the level of implementation of the global response as per the SRP.
Global response – Supply	% of countries requesting personal protective equipment that have received stockpiles	N/A	This indicator focuses on capacity to deploy supplies to countries during the event.
	# of companies/organizations actively participating in the PSCN	N/A	Indicates strength of the PSCN and breadth of coverage of private sector organizations at global and country levels related to WHO's operations.
Global response – R&D	% countries eligible to enroll in clinical trials that enrolled	TBD	This measure focuses on country collaboration and can serve to advocate for acceptance of multi-site clinical trials in countries where it was not feasible during the outbreak.
Country readiness – Capacity	Preparedness index & Operational readiness index (Using 18 different indicators from SPAR)	Level 1: <=30<br>Level 2: <=50%<br>Level 3: <=70%<br>Level 4: <=90%<br>Level 5: > 90%	Demonstrate the level of preparedness and operational readiness based on the implementation of IHR (2005) capacities. They are based on the objective assessments not on the functional evaluation. The findings should be triangulated with the other instruments like AAR and SIMex.

KPIs of the Policies and the procedures

Policy Number	Policy Title	Requirements	Tracking Process	How	Not met<br>0	Moderately met<br>1	Fully met<br>2
AN.OR-03-009	Risk communication and community engagement (C3)	the process for risk communication and community engagement during a health emergency	Observation<br>Interview<br>Auditing<br>Reports	5 Why<br>root cause analysis			
		the process for communication messages to be developed, approved and disseminated					
		The communication plans, strategies and procedures existed to guide risk communication during a health emergency					
		The coordination mechanisms (multi-sectoral risk communication and community engagement team, working group, task force) were adapted for risk communication and community engagement during the COVID-19 outbreak					
		Use the identified Platforms to identify risk communication with communities (Hotlines, university website, social media, student union groups)					
		rumours management and false information regarding COVID-19					
		Proactive precautions for populations at risks for COVID-19 (people with disabilities, pregnant women, elderly)					
		Challenges remained with respect to gender, equity and human right					

KPIs of the Policies and the procedures

Policy Number	Policy Title	Requirements	Tracking Process	How	Not met	Moderately met	Fully met
	Surveillance, case investigation and contact tracing	Apply the guidelines, SOPs and protocols	Observation<br>Interview<br>Auditing<br>Reports	5 Why<br>root cause analysis			
		Identify the process for data analysis					
		RRT and training on SOPs.					
		utilization of the resources (human/financial/material) to undertake warning during the COVID-19 outbreak					
		SOPs and protocol have been developed to detect cases, conduct contact tracing and monitor contacts					
		the legal framework for rapid response teams (RRTs)					
		Identify other mechanisms (EMTs, workers, university professors) besides RRTs used in the COVID-19 response					
		Consider gender, equity and human rights and disadvantaged subpopulations					
		Identify the guidelines, SOPs and protocols in place.					
		the case definitions of COVID-19 surveillance guidance					
		contact tracing and monitoring (Go Data)					
		epidemiological data management, analysis					
		Gender, equity and human rights and disadvantaged subpopulations					

KPIs of the Policies and the procedures

Policy Number	Policy Title	Requirements	Tracking Process	How	Not met	Moderately met	Fully met
	Identification of the Points of entry POEs	the emergency contingency plans, guidance for hospital referral, and SOP	Observation<br>Interview<br>Auditing<br>Reports	5 Why root cause analysis			
		the workforce training and equipment					
		health facilities, healthcare staff					
		Safe transportation for the students and staff					
		Safe entry for the students and staff					
		Safe transportation of suspected COVID-19					
		IPC measures					
		RRT trained on management of ill students and suspected cases					
		Consider gender, equity and human rights and disadvantaged subpopulations.					
		available plans and guidance materials for the COVID-19 response					
		Screening measures (thermometer, Rapid Antigen Test)					
		the coordination of information exchange between conveyance operators (frequency of					

KPIs of the Policies and the procedures

Policy Number	Policy Title	Requirements	Tracking Process	How	Not met	Moderately met	Fully met
	**Infection prevention and control (IPCs)**	Obligate to the SOPs and preparedness measure for protecting staff, students, and other frontline	Observation<br>Interview<br>Auditing<br>Reports	5 Why<br>root cause analysis			
		stockpiles of PPE for healthcare staff and other frontline workers					
		the effectiveness of the IPC					
		clean water supply					
		SOPs for waste management					
		human resources responsibility for IPC					
		managing and distributing of PPE					
		Legislations and advisories for mandatory IPC measures					
		IPC measures in confirmed case of COVID-19					

KPIs of the Policies and the procedures

October 6 University

Exams Policy KPIs

Policy Number	Policy Title	Requirements	Tracking Process	How	Not met	Moderately met	Fully met
	**Case management and knowledge sharing**	The Identification the criteria of COVID-19 suspected and confirmed patients	Observation<br>Interview<br>Auditing<br>Reports	5 Why<br>root cause analysis			
		The coordination of case management between healthcare facilities, private sectors and partners					
		the reassignment of medical staff					
		Surge capacity management					
		detect and to initiate the management and referral of potential COVID-19					
		The hazardous waste management instruction					
		IPC administrative control measures implementation					
		local networks for discussions on clinical management					

KPIs of the Policies and the procedures

Policy Number	Policy Title	Requirements	Tracking Process	How	Not met	Moderately met	Fully met
	Operational support and logistics in the management of supply chains and the workforce	The identification of the most risk supplies of losing stability	Observation<br>Interview<br>Auditing<br>Reports	5 Why<br>root cause analysis			
		the supply chain steps identification					
		Identification of the significant risk					
		scaling up operations and logistics in emergency					
		A prospective evaluation of the supplies and the suppliers					
		A retrospective evaluation for the supplies					
		Operational and logistics system management					
		The tracing process					
		plans and procedures established for the procurement of essential material for COVID-19 response,					

KPIs of the Policies and the procedures

Policy Number	Policy Title	Requirements	Tracking Process	How	Not met	Moderately met	Fully met
	Rapid Response Teams (RRTs) Rapid Response Teams (RRTs)	Follow the SOPS of active case finding and contact tracing	Observation<br>Interview<br>Auditing<br>Reports	5 Why root cause analysis			
		laboratory samples according to the WHO sampling checklist					
		Apply the occupational health and safety hazards, assess key risks, and outline measures.					
		Monitor the application of Infection Prevention and Control measures					
		Managing rumors and misinformation					
		Use the rumor log template for documenting rumors					
		Monitoring tools for assessing the efficiency and the effective measures in the context of COVID-19.					
		local networks for discussions on clinical management and mull practice					
		Follow the SOPS of active case finding and contact tracing					

IAR_ Presenting IAR results and follow-up action

جامعة ٦ أكتوبر
OCTOBER 6 UNIVERSITY

KPIs Score

KPI	App.Arts	Computer Sci	Medical Sci	Dentistry	Eco.& Manag.	Education	Engineering	Mass Comm	Medicine	Pharmacy	Physical Therapy	Tourism	Nursing	Lang.&translation
C3 Total (40)	39	39	39	35	39	39	39	39	38	37	38	33	32	33
Surveillance Total (45)	38	37	38	37	38	38	39	37	40	38	37	38	37	38
Identification of the Points of entry total (50)	47	47	47	43	47	47	47	47	47	45	46	47	47	47
Infection prevention and control total (95)	89	89	89	76	85	85	84	86	89	86	87	88	88	88
Case management and knowledge sharing total (40)	30	29	30	30	30	28	28	28	33	31	28	30	26	30
Op. support Total (45)	40	40	40	40	40	40	40	40	40	40	40	40	40	40
RRTs Total (40)	20	20	20	16	20	20	20	20	24	20	20	20	20	20
Grand total (355)	303	301	303	277	299	297	297	297	311	297	296	296	290	296
KPI (%)	App.Arts	Computer Sci	Medical Sci	Dentistry	Eco.& Manag.	Education	Engineering	Mass Comm	Medicine	Pharmacy	Physical Therapy	Tourism	Nursing	Lang.&translation
C3 Total	98	98	98	88	98	98	98	98	95	93	95	83	80	83
Surveillance Total	84	82	84	82	84	84	87	82	89	84	82	84	82	84
Identification of the Points of entry total	94	94	94	86	94	94	94	94	94	90	92	94	94	94
Infection prevention and control total	94	94	94	80	89	89	88	91	94	91	92	93	93	93
Case management\	75	73	75	75	75	70	70	70	83	78	70	75	65	75
Op. support Total	89	89	89	89	89	89	89	89	89	89	89	89	89	89
RRTs Total	50	50	50	40	50	50	50	50	60	50	50	50	50	50
Grand total	85	85	85	78	84	84	84	84	88	84	83	83	82	83
	Level 4: <=90%	Level 4: <=90%	Level 4: <=90%	Level 1: <=30	Level 4: <=90%	Level 4: <=90%	Level 4: <=90%	Level 4: <=90%	Level 4: <=90%	Level 4: <=90%	Level 4: <=90%	Level 4: <=90%	Level 4: <=90%	Level 4: <=90%

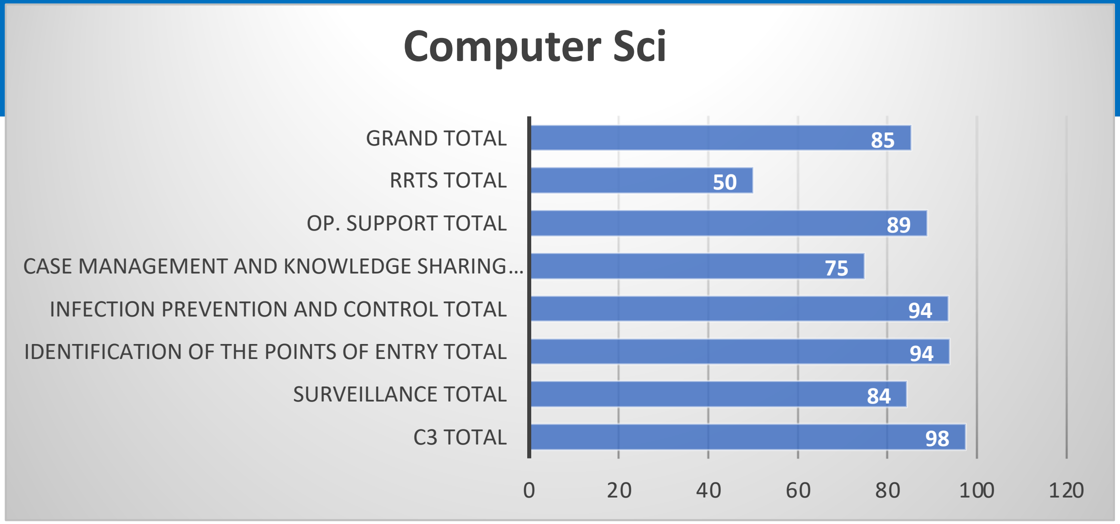
Computer Sci
GRAND TOTAL 85
RRTS TOTAL 50
OP. SUPPORT TOTAL 89
CASE MANAGEMENT AND KNOWLEDGE SHARING... 75
INFECTION PREVENTION AND CONTROL TOTAL 94
IDENTIFICATION OF THE POINTS OF ENTRY TOTAL 94
SURVEILLANCE TOTAL 84
C3 TOTAL 98
0 20 40 60 80 100 120

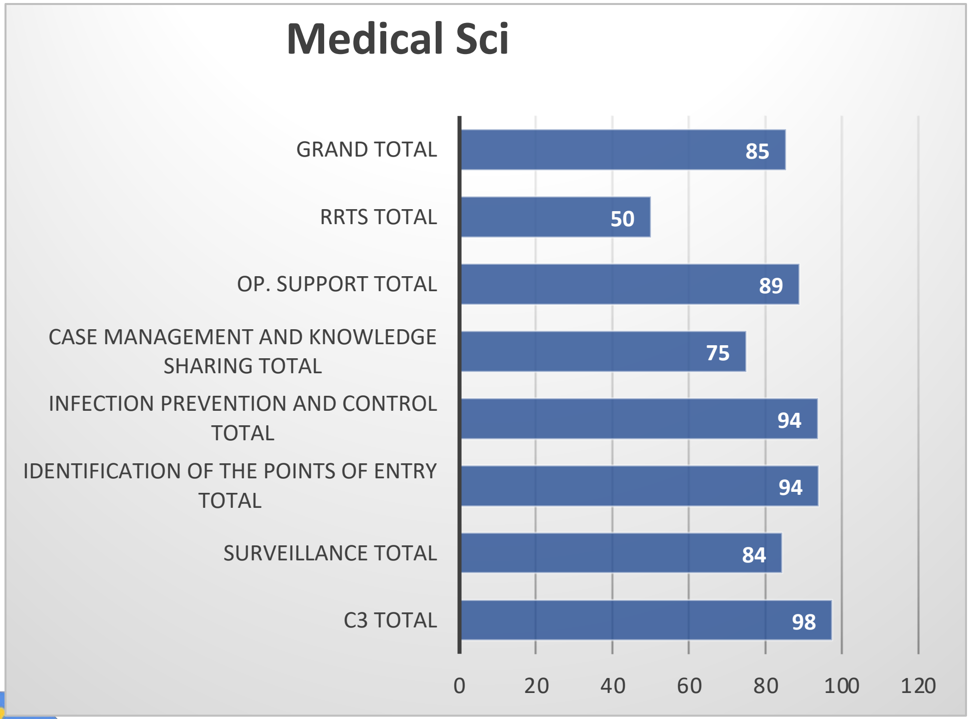
Medical Sci
GRAND TOTAL 85
RRTS TOTAL 50
OP. SUPPORT TOTAL 89
CASE MANAGEMENT AND KNOWLEDGE SHARING TOTAL 75
INFECTION PREVENTION AND CONTROL TOTAL 94
IDENTIFICATION OF THE POINTS OF ENTRY TOTAL 94
SURVEILLANCE TOTAL 84
C3 TOTAL 98
0 20 40 60 80 100 120

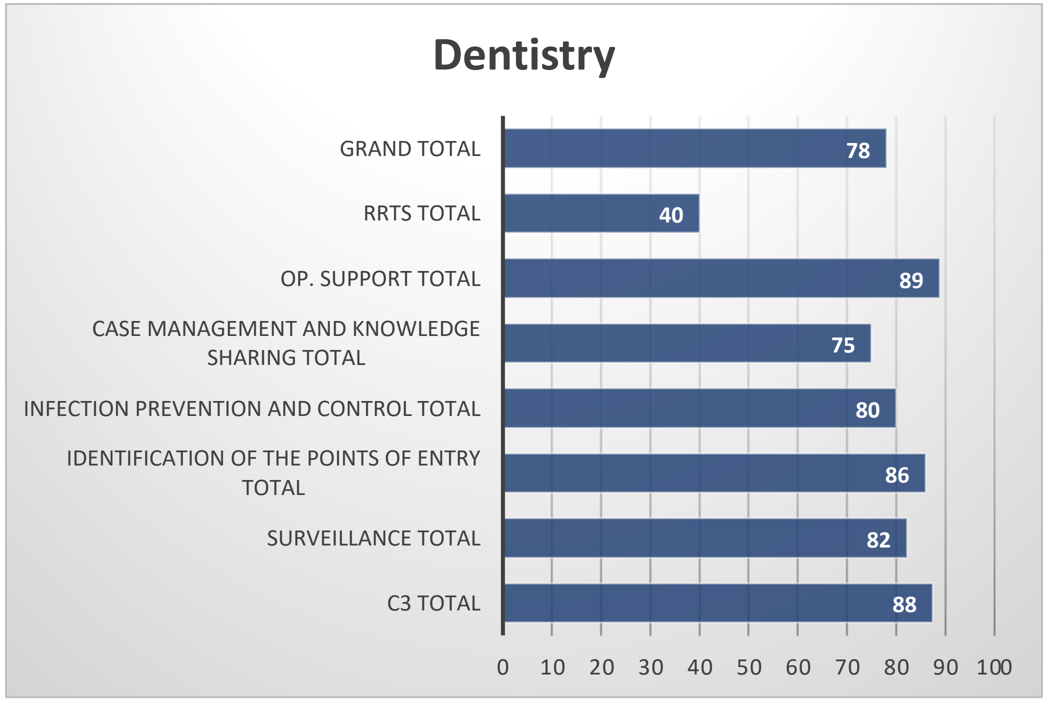
Dentistry
GRAND TOTAL 78
RRTS TOTAL 40
OP. SUPPORT TOTAL 89
CASE MANAGEMENT AND KNOWLEDGE SHARING TOTAL 75
INFECTION PREVENTION AND CONTROL TOTAL 80
IDENTIFICATION OF THE POINTS OF ENTRY TOTAL 86
SURVEILLANCE TOTAL 82
C3 TOTAL 88
0 10 20 30 40 50 60 70 80 90 100

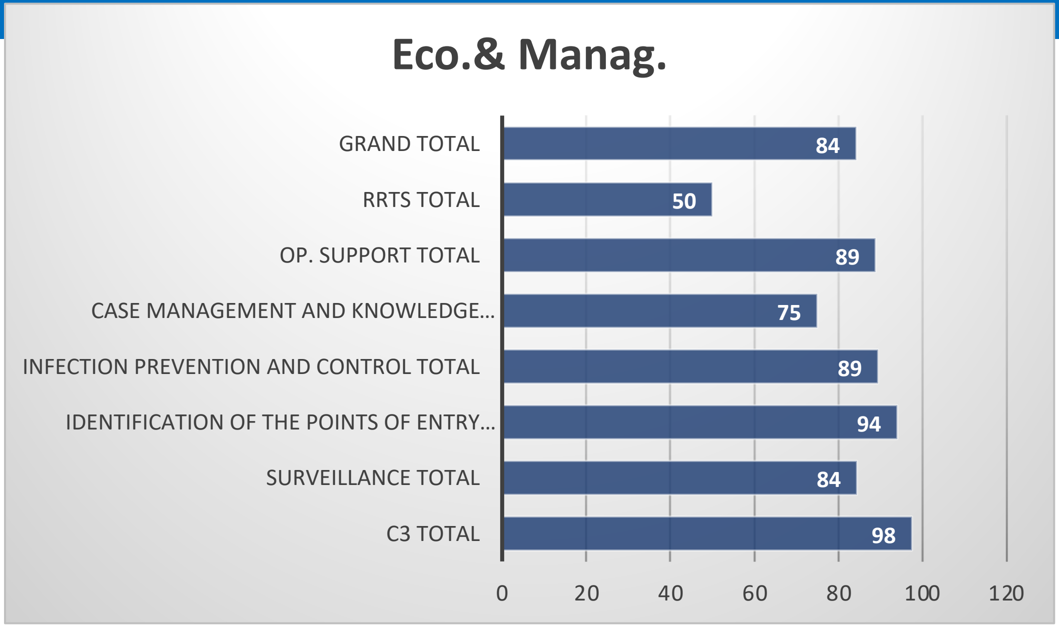
Eco.& Manag.
GRAND TOTAL
RRTS TOTAL
OP. SUPPORT TOTAL
CASE MANAGEMENT AND KNOWLEDGE...
INFECTION PREVENTION AND CONTROL TOTAL
IDENTIFICATION OF THE POINTS OF ENTRY...
SURVEILLANCE TOTAL
C3 TOTAL
84
50
89
75
89
94
84
98
0
20
40
60
80
100
120

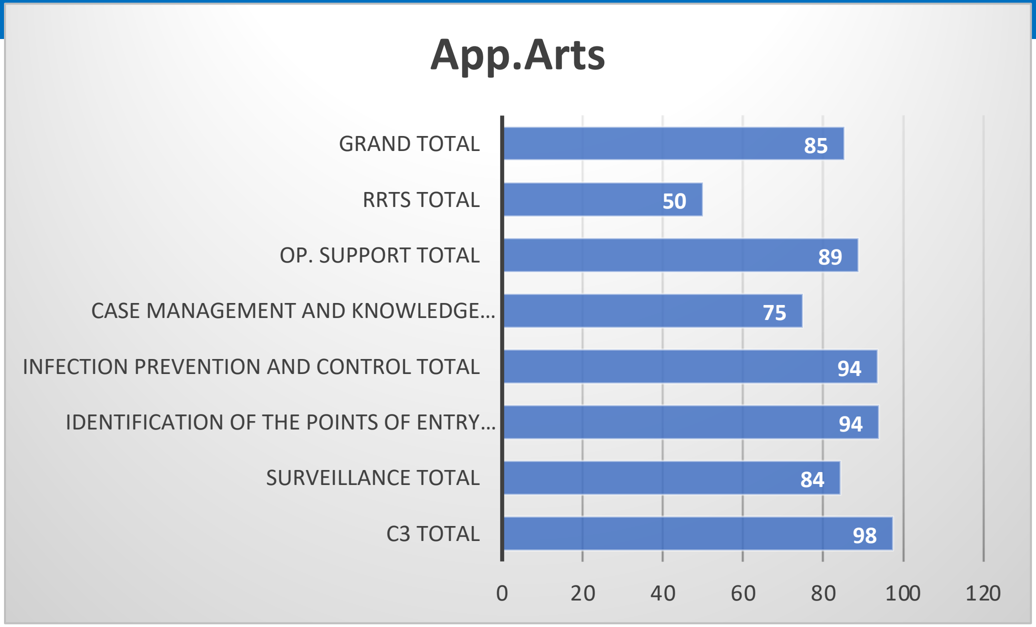
App.Arts
GRAND TOTAL
RRTS TOTAL
OP. SUPPORT TOTAL
CASE MANAGEMENT AND KNOWLEDGE...
INFECTION PREVENTION AND CONTROL TOTAL
IDENTIFICATION OF THE POINTS OF ENTRY...
SURVEILLANCE TOTAL
C3 TOTAL
85
50
89
75
94
94
84
98
0
20
40
60
80
100
120

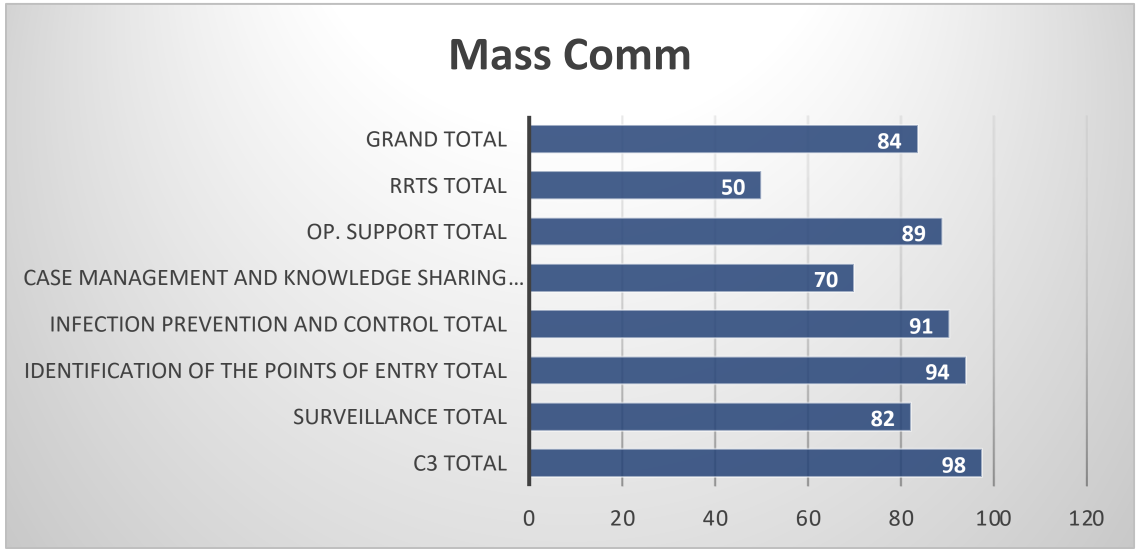
Mass Comm
GRAND TOTAL
RRTS TOTAL
OP. SUPPORT TOTAL
CASE MANAGEMENT AND KNOWLEDGE SHARING...
INFECTION PREVENTION AND CONTROL TOTAL
IDENTIFICATION OF THE POINTS OF ENTRY TOTAL
SURVEILLANCE TOTAL
C3 TOTAL
84
50
89
70
91
94
82
98
0
20
40
60
80
100
120

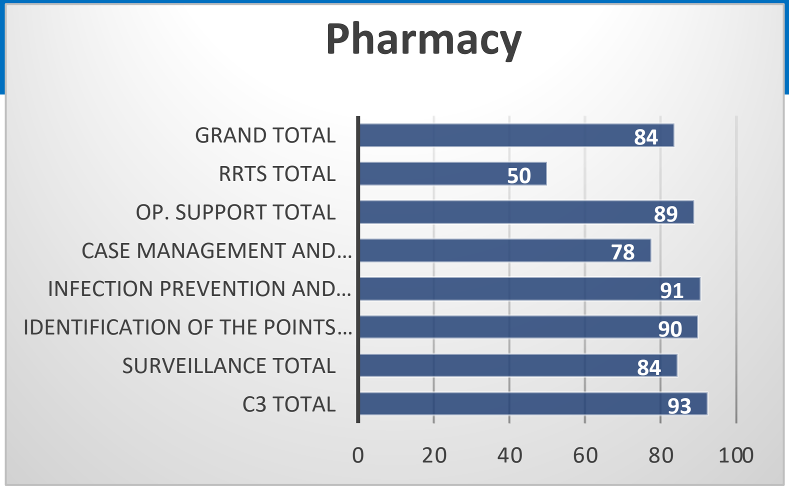
Pharmacy
GRAND TOTAL
RRTS TOTAL
OP. SUPPORT TOTAL
CASE MANAGEMENT AND...
INFECTION PREVENTION AND...
IDENTIFICATION OF THE POINTS...
SURVEILLANCE TOTAL
C3 TOTAL
84
50
89
78
91
90
84
93
0
20
40
60
80
100

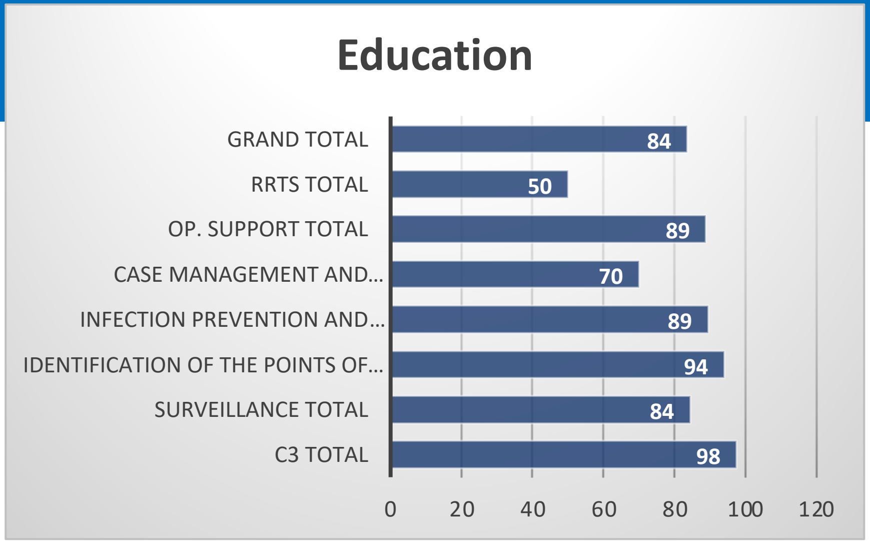
Education
GRAND TOTAL
RRTS TOTAL
OP. SUPPORT TOTAL
CASE MANAGEMENT AND...
INFECTION PREVENTION AND...
IDENTIFICATION OF THE POINTS OF...
SURVEILLANCE TOTAL
C3 TOTAL
84
50
89
70
89
94
84
98
0
20
40
60
80
100
120

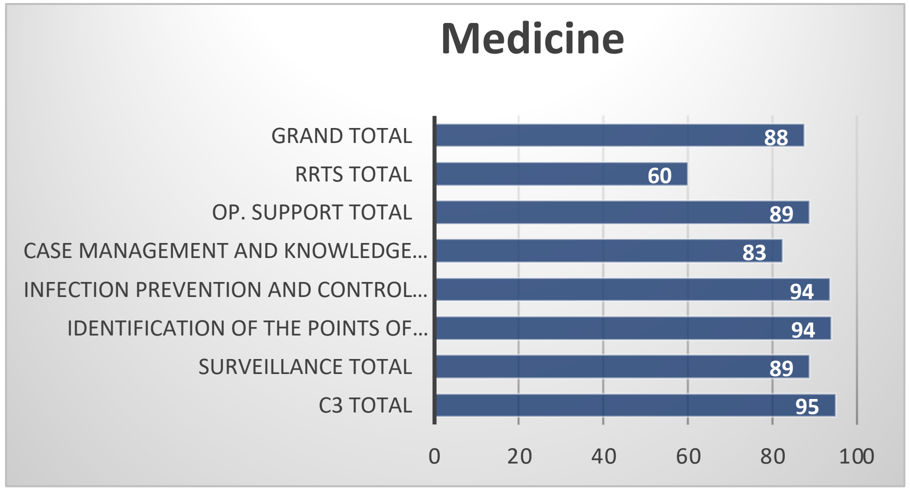
Medicine
GRAND TOTAL
RRTS TOTAL
OP. SUPPORT TOTAL
CASE MANAGEMENT AND KNOWLEDGE...
INFECTION PREVENTION AND CONTROL...
IDENTIFICATION OF THE POINTS OF...
SURVEILLANCE TOTAL
C3 TOTAL
88
60
89
83
94
94
89
95
0
20
40
60
80
100

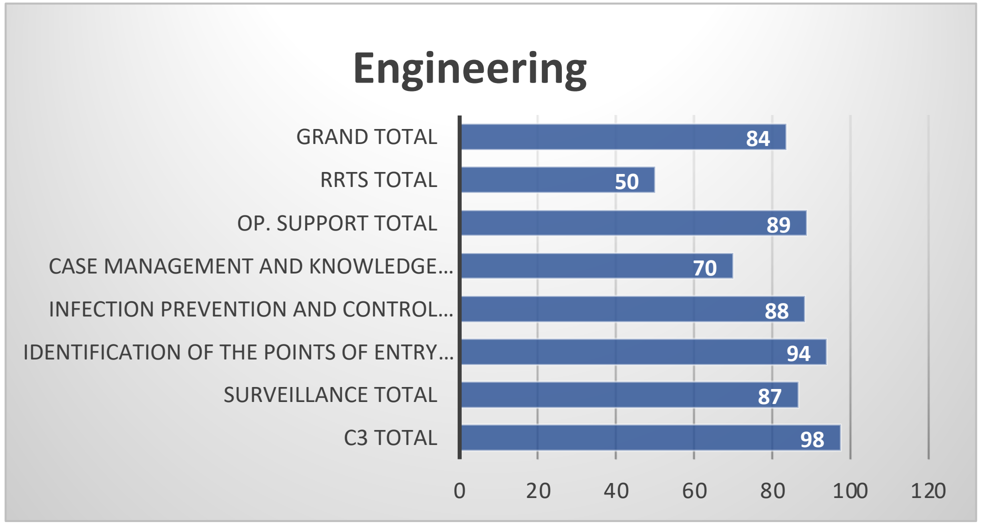
Engineering
GRAND TOTAL
RRTS TOTAL
OP. SUPPORT TOTAL
CASE MANAGEMENT AND KNOWLEDGE...
INFECTION PREVENTION AND CONTROL...
IDENTIFICATION OF THE POINTS OF ENTRY...
SURVEILLANCE TOTAL
C3 TOTAL
84
50
89
70
88
94
87
98
0
20
40
60
80
100
120

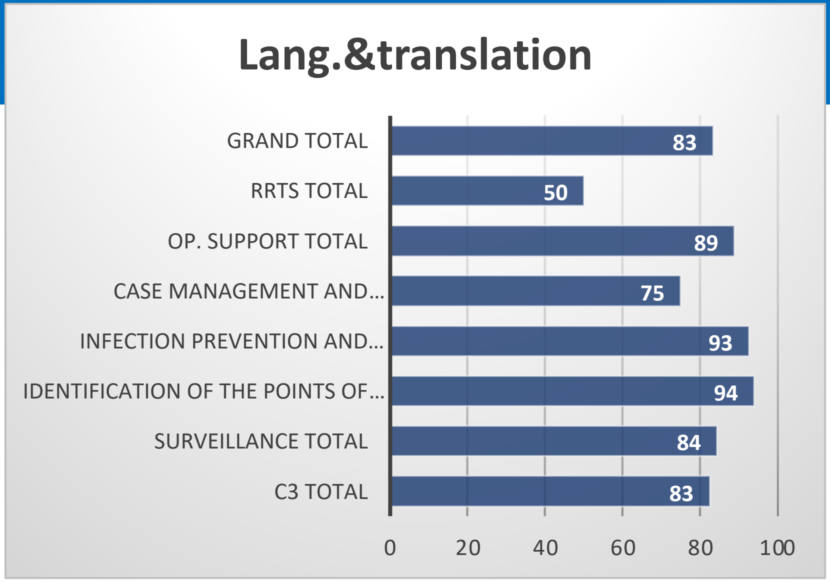
Lang.&translation
GRAND TOTAL
RRTS TOTAL
OP. SUPPORT TOTAL
CASE MANAGEMENT AND...
INFECTION PREVENTION AND...
IDENTIFICATION OF THE POINTS OF...
SURVEILLANCE TOTAL
C3 TOTAL
83
50
89
75
93
94
84
83
0
20
40
60
80
100

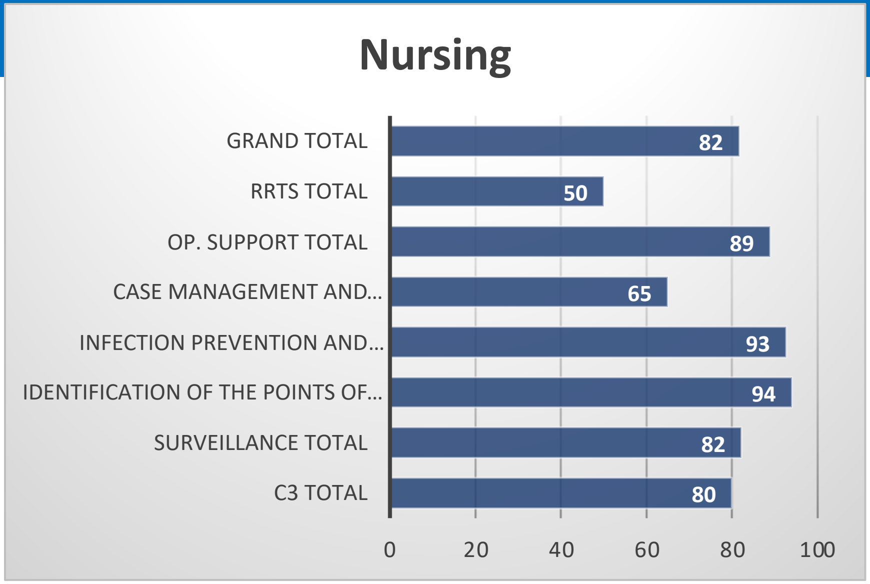
Nursing
GRAND TOTAL
RRTS TOTAL
OP. SUPPORT TOTAL
CASE MANAGEMENT AND...
INFECTION PREVENTION AND...
IDENTIFICATION OF THE POINTS OF...
SURVEILLANCE TOTAL
C3 TOTAL
82
50
89
65
93
94
82
80
0
20
40
60
80
100

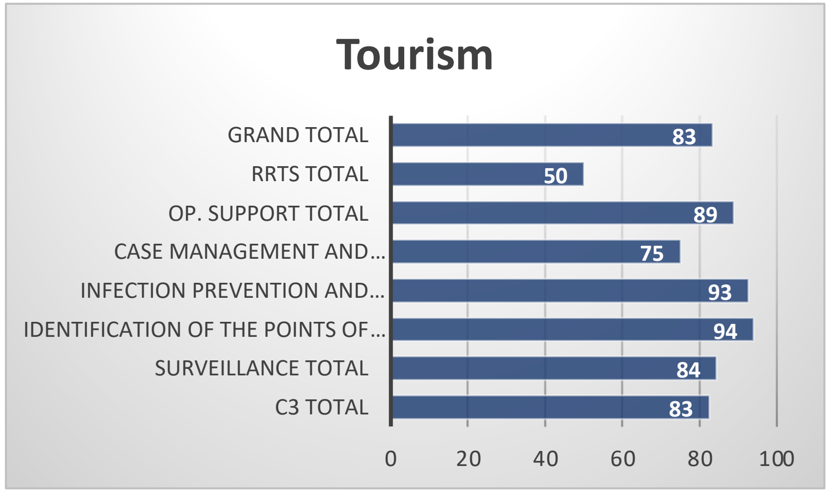
Tourism
GRAND TOTAL
RRTS TOTAL
OP. SUPPORT TOTAL
CASE MANAGEMENT AND...
INFECTION PREVENTION AND...
IDENTIFICATION OF THE POINTS OF...
SURVEILLANCE TOTAL
C3 TOTAL
83
50
89
75
93
94
84
83
0
20
40
60
80
100

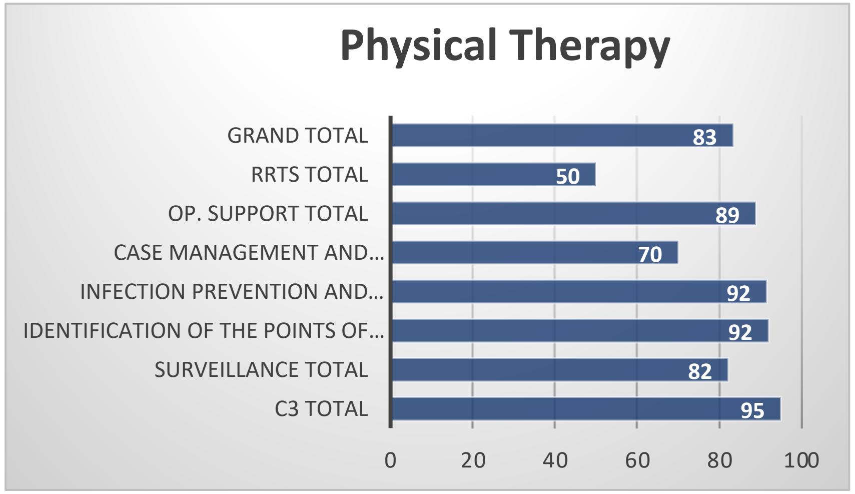
Physical Therapy
GRAND TOTAL
RRTS TOTAL
OP. SUPPORT TOTAL
CASE MANAGEMENT AND...
INFECTION PREVENTION AND...
IDENTIFICATION OF THE POINTS OF...
SURVEILLANCE TOTAL
C3 TOTAL
83
50
89
70
92
92
82
95
0
20
40
60
80
100

Post-event phase (AAR)

INTRA-ACTION REVIEW OVERVIEW

Introduction: **Response plan and actual timeline of the response**

STEP 1

Step 1: **What went well? What went less well? Why?**

STEP 2

Step 2: **What can we do to improve the COVID-19 response?**

STEP 3

Step 3: **The Way Forward**

STEP 1

Step 1 : **What went well? What went less well? Why?**

STRENGTHS

&

CHALLENGES

of the response

CONTRIBUTING FACTORS

(root cause analysis
see next slide)

STEP 1

Step 1 : **What went well? What went less well? Why?**

CHALLENGES

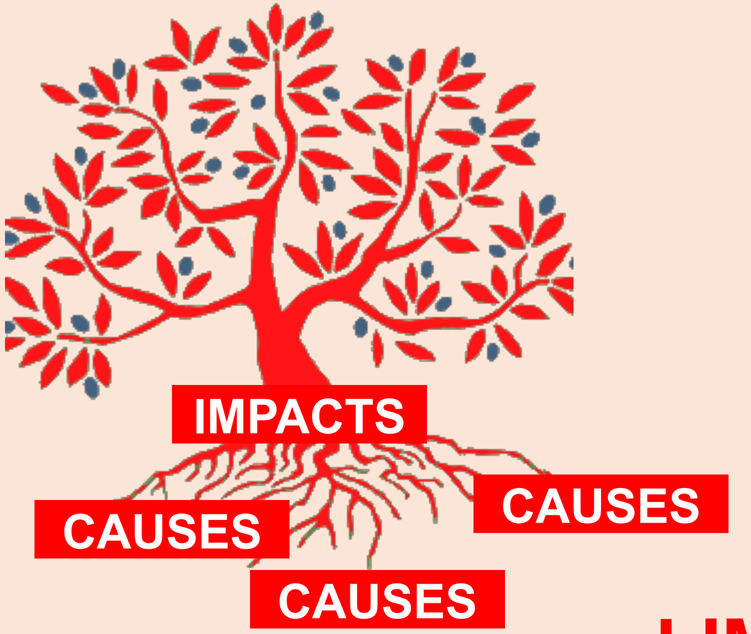

5 WHY's

STRENGTHS

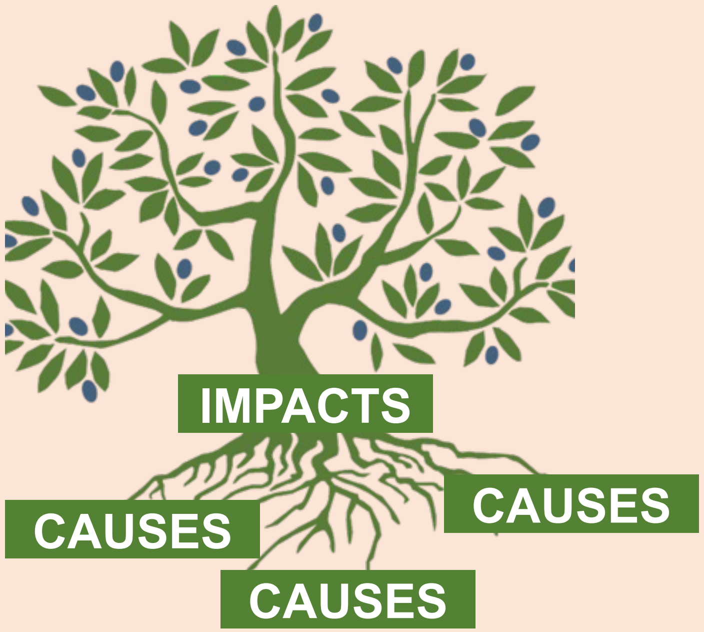

LIMITING & ENABLING FACTORS

STEP 1

Step 1 : **What went well? What went less well? Why?**

1. Using the trigger questions, identify the challenges and best practices during the response
2. For each challenge and best practice, identify the impact this has had on the response during the period under review
3. For each challenge and best practice, identify limiting factors (for challenges) and facilitating factors (for best practices)
4. Identify no more than 6 key challenges and 6 key best practices

STEP 1

Step 1 : **What went well? What went less well? Why?**

Best Practices/Strengths	Impact(s)	Enabling factors
Regular cross-border coordination meetings	Improved coordination and sharing of information for the early detection of suspected/ confirmed cases and for monitoring contacts	• Relationship had been established prior to the response<br>• Willingness of all stakeholders to undertake regular meetings<br>• Political and financial support from central level

Challenges	Impact(s)	Limiting Factors
Coordination at local level ineffective	• Response not coordinated between partners, health authorities and the central level<br>• Duplication of activities and effort	• Lack of a plan for district level coordination<br>• Partners not participating in coordination meetings

EXAMPLE

Step 1 : **What went well? What went less well? Why?**

STEP 1

Important definitions

Best practice:
Something that was done during the COVID-19 response that improved performance or had a positive impact

Examples:

- Development of new SOPs for COVID-19 diagnosis
- Organization of cross-border meetings during the COVID-19 response to facilitate better coordination

STEP 1

Step 1 : **What went well? What went less well? Why?**

Important definitions

Challenge:

Job, duty or situation that is difficult during the COVID-19 response because you must use a lot of effort, determination, and skill in order to be successful.

Examples:

- Lack of coordinated communication between MoH and partners.
- Limited capacity for COVID-19 testing at subnational level.

STEP 1

Step 1 : **What went well? What went less well? Why?**

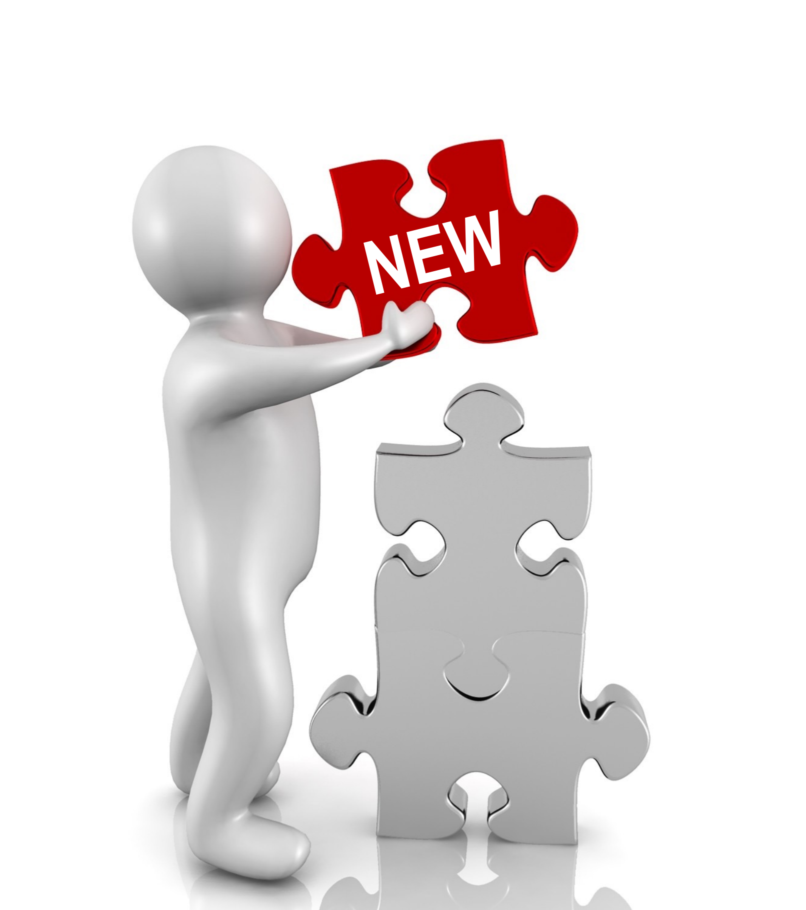

Remember the objective is:

To identify the best practices and the key challenges

but also

To identify and institutionalize
new capacities
Developed so far during the response

e.g. new SOP, new equipment purchased, new skills learned…etc.

INTRA-ACTION REVIEW OVERVIEW

Introduction: **Response plan and actual timeline of the response**

STEP 1

Step 1: **What went well? What went less well? Why?**

STEP 2

Step 2: **What can we do to improve the COVID-19 response?**

STEP 3

Step 3: **The Way Forward**

Step 2 : **What can we do to improve the COVID-19 response?**

STEP 2

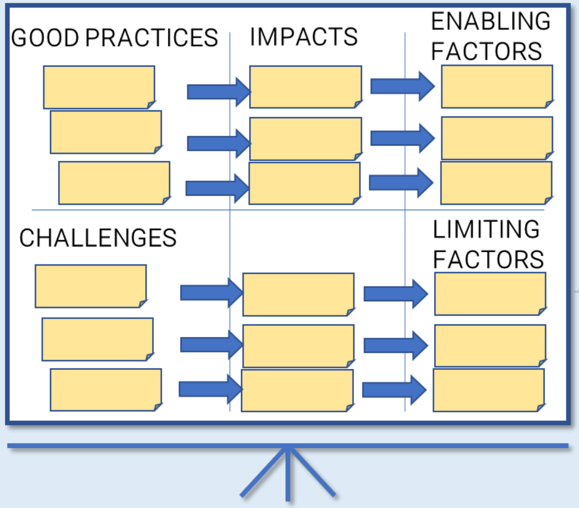

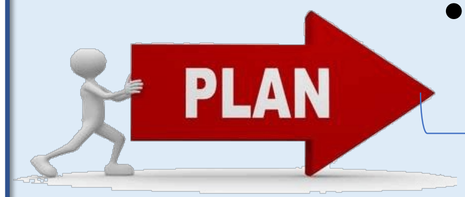

- Institutionalize best practices
- Address challenges

Development of specific activities:

- to build on enabling factors
- to address the limiting factors

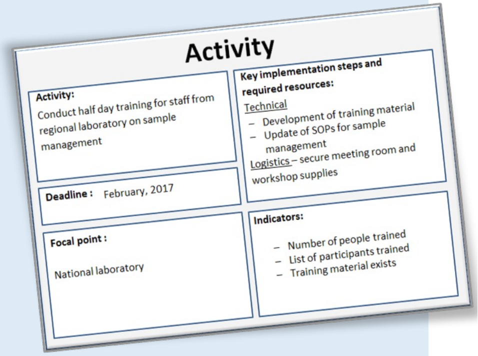

Step 2 : **What can we do to improve the COVID-19 response?**

STEP 2

EXAMPLE

Activity

Activity:

Conduct half day training for staff from regional laboratory on sample management

Deadline : February 1st, 2021

Focal point :

National laboratory

Key implementation steps and required resources:

Technical
- % of SOPs updated
- Development of training materials

Logistics
- Secure meeting room and workshop supplies

Indicators:

- Percentage of people trained who can manage properly samples

- All activities need to be practical and realistic
- Several activities might be necessary to address a single challenge or a best practice
- Not all best practices or challenges need an activity

STEP 2

Step 2 : **What can we do to improve the COVID-19 response?**

Refer to the challenges, best practice, impact and factors to help identify key activities to overcome challenges and institutionalize best practices.

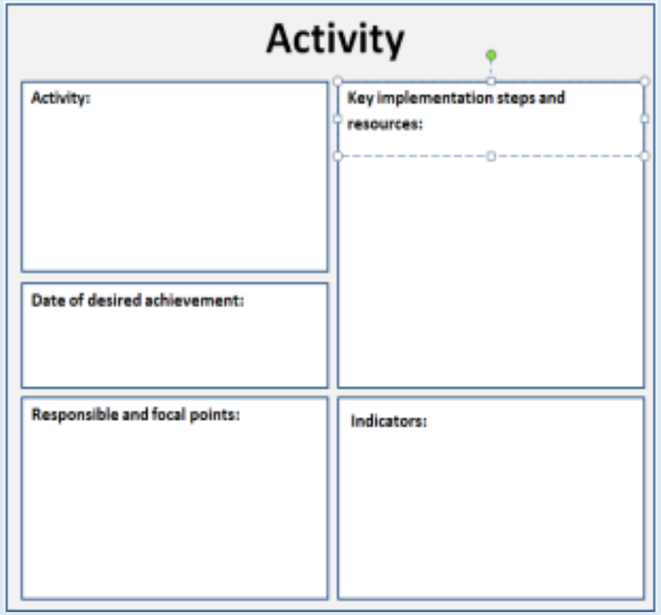
Activity

Activity:	Key implementation steps and resources:
Date of desired achievement:	
Responsible and focal points:	Indicators:

INTRA-ACTION REVIEW OVERVIEW

Introduction: **Response plan and actual timeline of the response**

STEP 1

Step 1: **What went well? What went less well? Why?**

STEP 2

Step 2: **What can we do to improve the COVID-19 response?**

STEP 3

Step 3: **The Way Forward**

STEP 3

Step 3 : **The Way Forward**

In the plenary session, discuss and agree on the following:

- Identification of:
  - what can be addressed immediately to improve the ongoing response;
  - what can be done in the mid and long-term to improve response to the next waves of the COVID-19 outbreak.
- Establishment of an Intra-Action Review Follow-up team
- Process to document progress in implementing the recommendations
- Approach to ensure engagement of senior leadership

IAR _Participant feedback form

October 6 University COVID-19 INTRA-ACTION REVIEW (IAR) – PARTICIPANT FEEDBACK FORM results

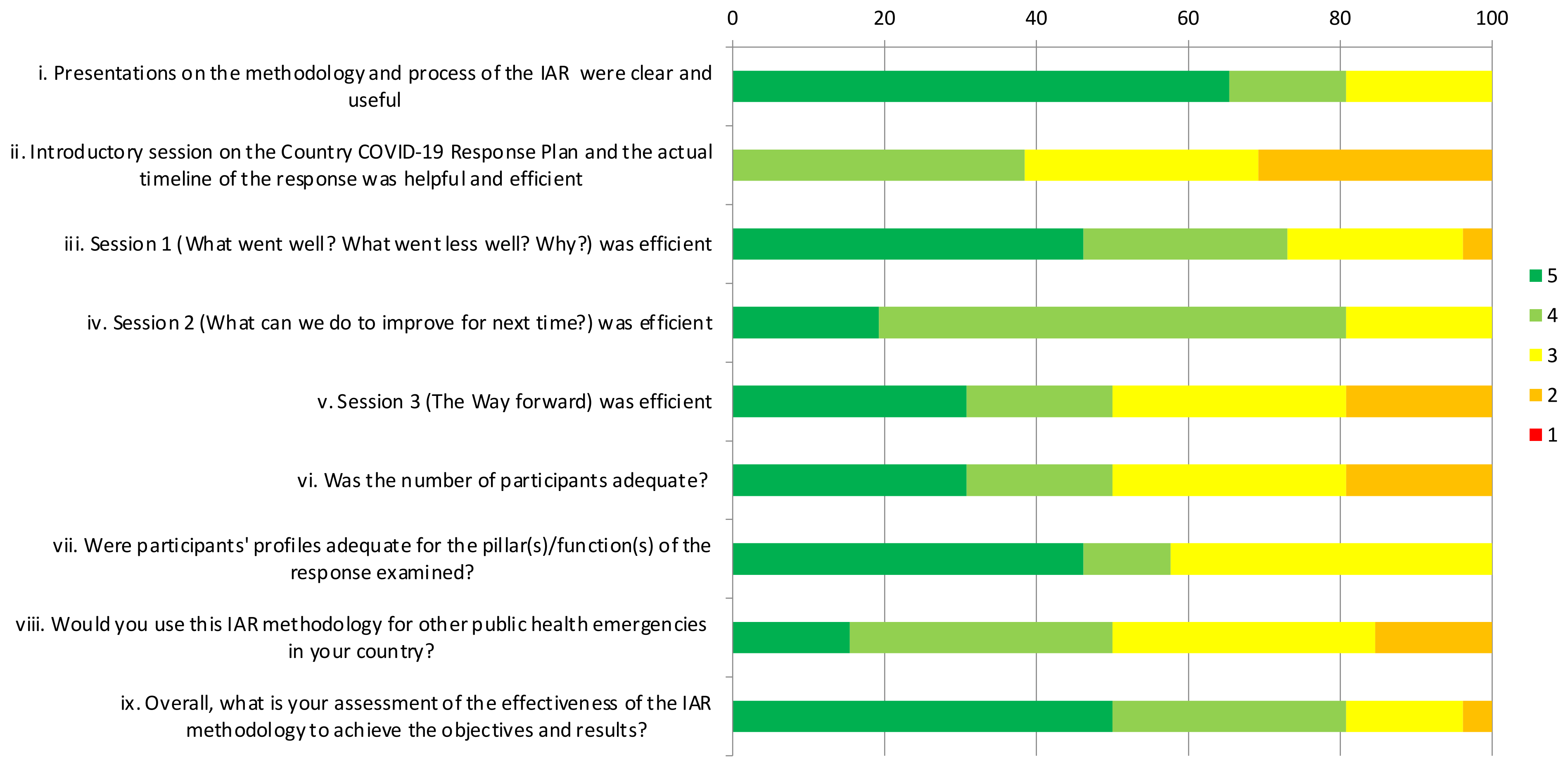

Q3. On a scale of 1 (not at all likely) to 5 (extremely likely), to what extent do you think the results of the IAR can contribute to:

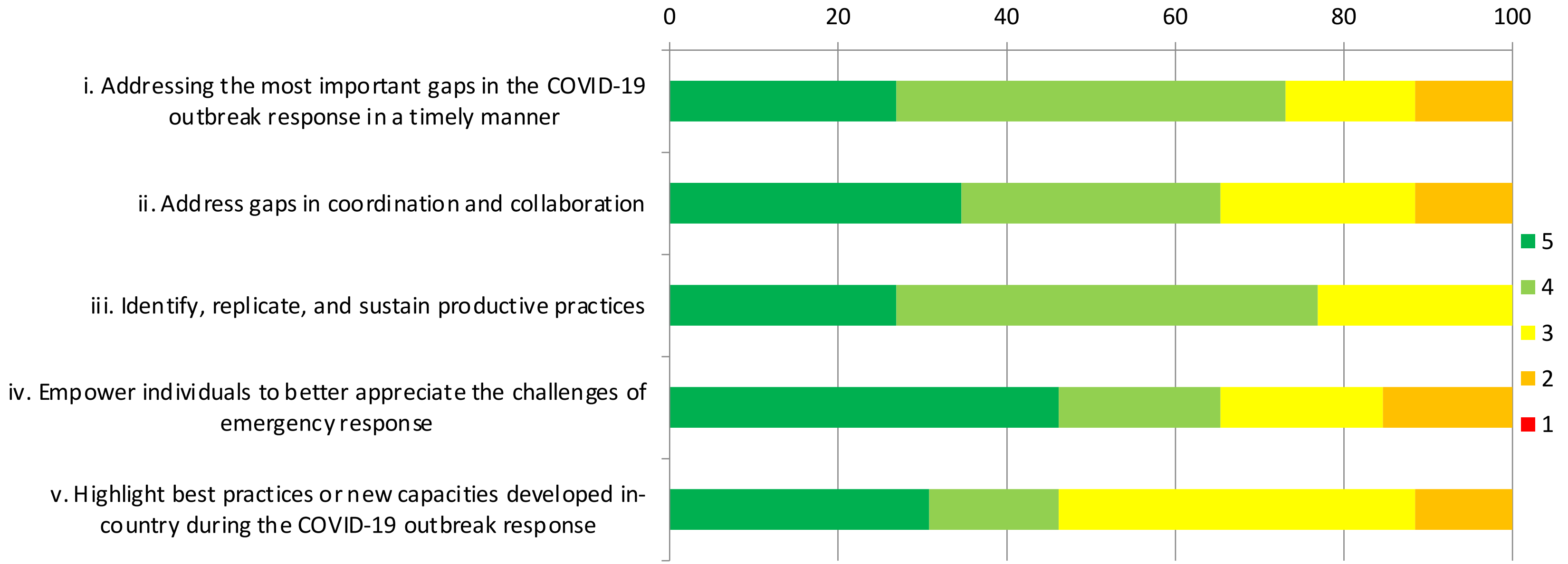

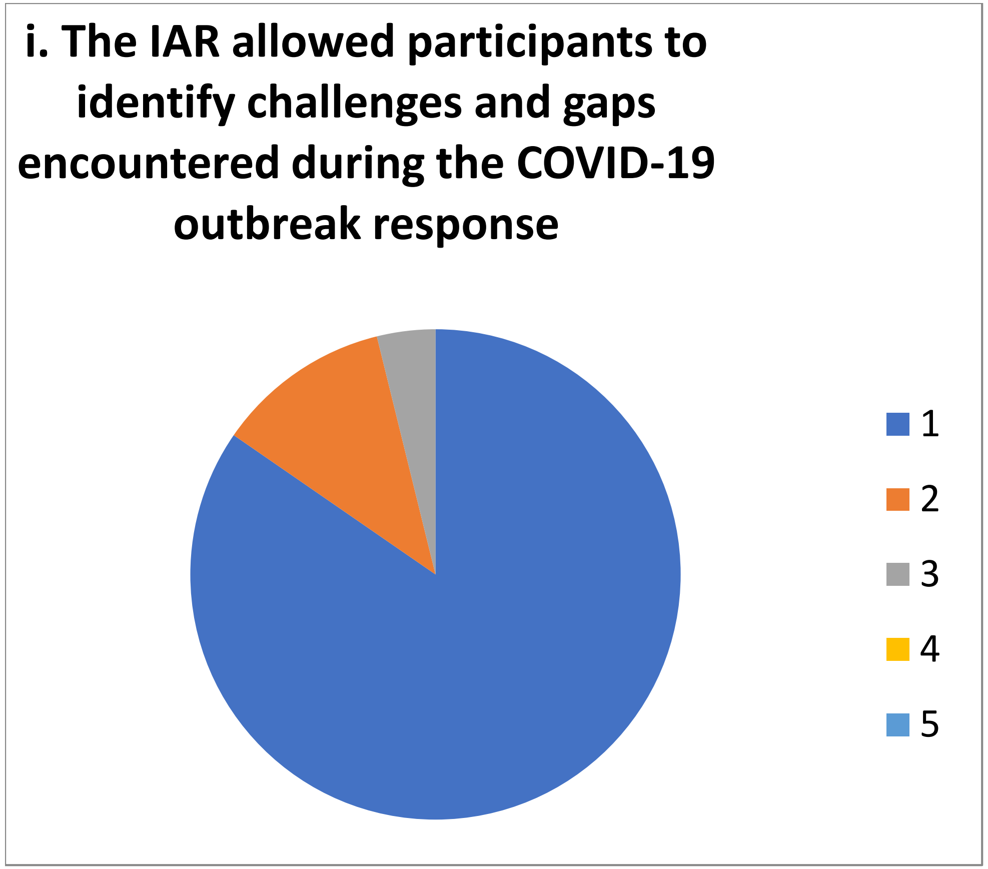
i. The IAR allowed participants to identify challenges and gaps encountered during the COVID-19 outbreak response
1
2
3
4
5

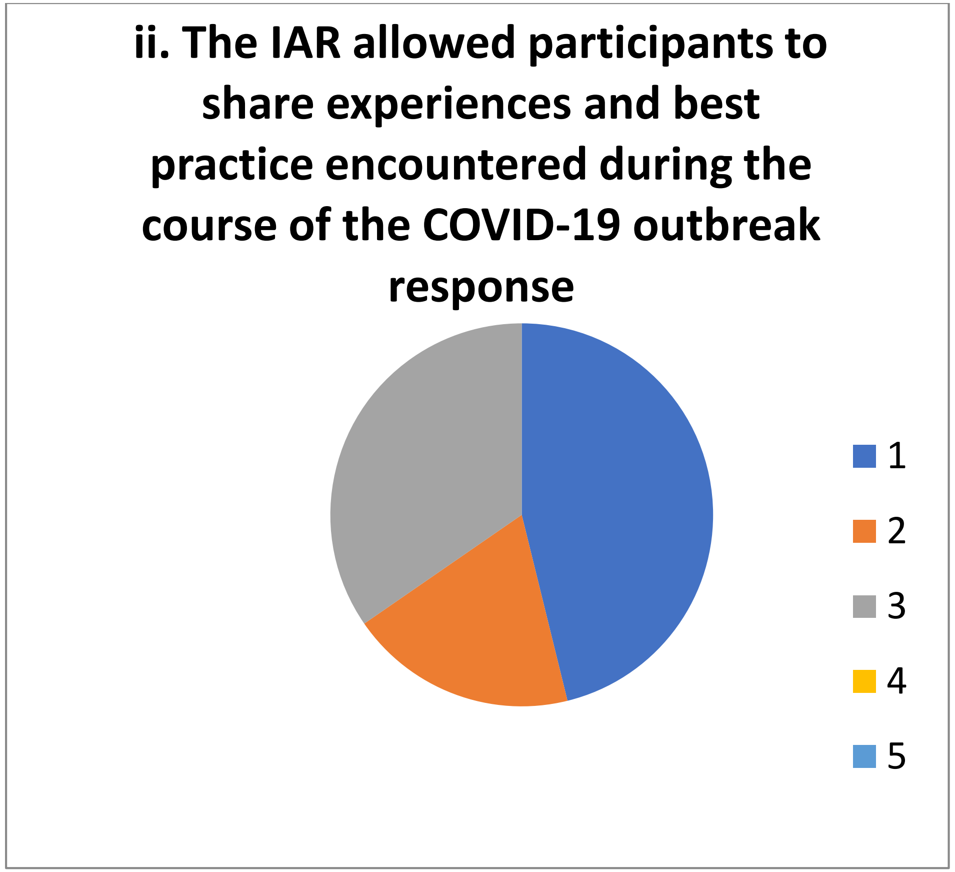
ii. The IAR allowed participants to share experiences and best practice encountered during the course of the COVID-19 outbreak response
1
2
3
4
5

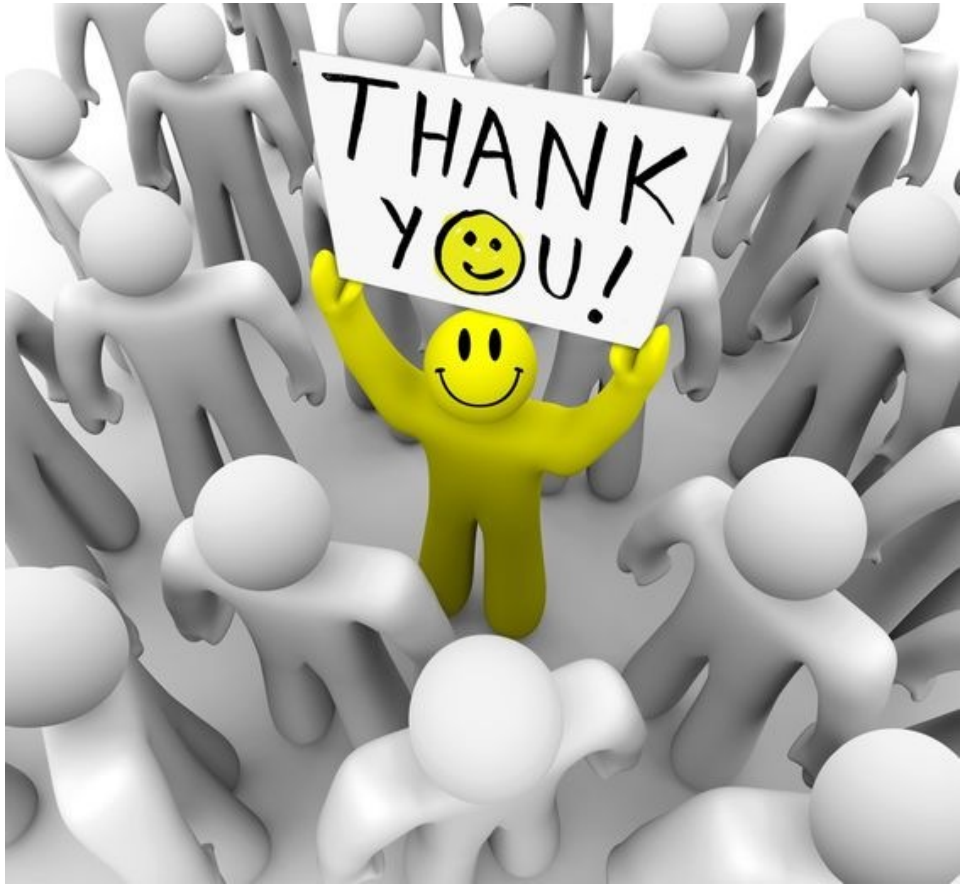

Thank for your contribution to saving lives!
